# Integrity of Narrow Epithelial Tubes in the *C*. *elegans* Excretory System Requires a Transient Luminal Matrix

**DOI:** 10.1371/journal.pgen.1006205

**Published:** 2016-08-02

**Authors:** Hasreet K. Gill, Jennifer D. Cohen, Jesus Ayala-Figueroa, Rachel Forman-Rubinsky, Corey Poggioli, Kevin Bickard, Jean M. Parry, Pu Pu, David H. Hall, Meera V. Sundaram

**Affiliations:** 1 Department of Genetics, Perelman School of Medicine, University of Pennsylvania, Philadelphia, Pennsylvania, United States of America; 2 Department of Biology, Georgian Court University, Lakewood, New Jersey, United States of America; 3 Department of Neuroscience, Albert Einstein College of Medicine, Bronx, New York, United States of America; University of California San Diego, UNITED STATES

## Abstract

Most epithelial cells secrete a glycoprotein-rich apical extracellular matrix that can have diverse but still poorly understood roles in development and physiology. Zona Pellucida (ZP) domain glycoproteins are common constituents of these matrices, and their loss in humans is associated with a number of diseases. Understanding of the functions, organization and regulation of apical matrices has been hampered by difficulties in imaging them both *in vivo* and *ex vivo*. We identified the PAN-Apple, mucin and ZP domain glycoprotein LET-653 as an early and transient apical matrix component that shapes developing epithelia in *C*. *elegans*. LET-653 has modest effects on shaping of the vulva and epidermis, but is essential to prevent lumen fragmentation in the very narrow, unicellular excretory duct tube. We were able to image the transient LET-653 matrix by both live confocal imaging and transmission electron microscopy. Structure/function and fluorescence recovery after photobleaching studies revealed that LET-653 exists in two separate luminal matrix pools, a loose fibrillar matrix in the central core of the lumen, to which it binds dynamically via its PAN domains, and an apical-membrane-associated matrix, to which it binds stably via its ZP domain. The PAN domains are both necessary and sufficient to confer a cyclic pattern of duct lumen localization that precedes each molt, while the ZP domain is required for lumen integrity. Ectopic expression of full-length LET-653, but not the PAN domains alone, could expand lumen diameter in the developing gut tube, where LET-653 is not normally expressed. Together, these data support a model in which the PAN domains regulate the ability of the LET-653 ZP domain to interact with other factors at the apical membrane, and this ZP domain interaction promotes expansion and maintenance of lumen diameter. These data identify a transient apical matrix component present prior to cuticle secretion in *C*. *elegans*, demonstrate critical roles for this matrix component in supporting lumen integrity within narrow bore tubes such as those found in the mammalian microvasculature, and reveal functional importance of the evolutionarily conserved ZP domain in this tube protecting activity.

## Author Summary

Most organs in the body are made up of networks of tubes that transport fluids or gases. These tubes come in many different sizes and shapes, with some narrow capillaries being only one cell in diameter. As tubes develop and take their final shapes, they secrete various glycoproteins into their hollow interior or lumen. The functions of these luminal proteins are not well understood, but there is increasing evidence that they are important for lumen shaping and that their loss can contribute to diseases such as cardiovascular disease and chronic kidney disease. Through studies of the nematode *C*. *elegans*, we identified a luminal glycoprotein, LET-653, that is transiently expressed in multiple developing tube types but is particularly critical to maintain integrity of the narrowest, unicellular tubes. We identified protein domains that direct LET-653 to specific apical matrix compartments and mediate its oscillatory pattern of lumen localization. Furthermore, we showed that the LET-653 tube-protecting activity depends on a Zona Pellucida (ZP) domain similar to that found in the mammalian egg-coat and in many other luminal or sensory matrix proteins involved in human disease.

## Introduction

Most epithelial and endothelial tube cells secrete an apical extracellular matrix (aECM) or glycocalyx that lines the tube lumen and consists of a complex mix of gel-forming and fibril-forming glycoproteins, including both secreted and transmembrane proteoglycans, mucins, and zona pellucida (ZP) domain proteins [1–4]. There is increasing evidence that this aECM plays important developmental roles in shaping epithelial tubes [5–8]. For example, CD34/podocalyxin-family sialomucins are among the earliest markers of apical identity on developing lumens in vertebrates [9], and are important for vascular, lymphatic, kidney and gut tube integrity in mice [10–12], where they appear to play both anti-adhesive and signaling roles [13–17]. Proteoglycans or mucin-type glycoproteins expand lumen diameter in the sea urchin archenteron [18], *C*. *elegans* vulva [19] and in the Drosophila hindgut [20] and retina [21], consistent with a model in which hydration-mediated expansion of a gel-like aECM generates intraluminal pressure to drive circumferential lumen enlargement. A transient luminal cable consisting of the carbohydrate chitin and various ZP-domain proteins is required for uniform lumen expansion and/or tube integrity in developing Drosophila tracheal tubes [22–24], and has been proposed to function as a scaffold that connects to the apical membrane and resists and evenly distributes expansion forces generated by secretion-driven membrane growth [25]. ZP-domain proteins are also found within luminal matrices of the vertebrate vascular system, kidney and gut, and are mutated in diseases affecting the integrity of these tubes [26–29]. While these examples highlight the diverse and important roles that specific aECM components can play, we still have a very limited understanding of aECM composition in most tubes, or of how different aECM components interact and work together to influence tube shaping and maintenance.

We are using the *C*. *elegans* excretory system as a model to identify factors important for shaping and maintaining small, unicellular tubes. Unicellular tubes have an intracellular lumen and can be seamed (sealed by an autocellular junction) or seamless (lacking junction along the lumen) [30]. Unicellular tubes are prevalent in capillary beds of the mammalian microvasculature [31], and defects in their organization or maintenance may be associated with cardiovascular disease and stroke [32–35]. Narrower tubes may be particularly sensitive to defects in the aECM, or may have unique aECM components or organization that is tailored to their unique biophysical requirements [10,24,32,36].

*C*. *elegans* unicellular excretory tubes develop in the context of a luminal aECM that has been visualized by electron microscopy but characterized to only a limited extent [37,38]. This early aECM appears to differ molecularly between the largest and most internal excretory tube (the excretory canal cell) vs. the two smaller external excretory tubes (the excretory duct and pore cells). The duct and pore tubes, like all other external epithelia, eventually become lined with a collagenous cuticle that forms at the end of embryogenesis [39,40]. Prior to and during cuticle formation, external epithelia express the apically-localized transmembrane proteins LET-4 and EGG-6, members of the extracellular leucine-rich repeat only (eLRRon) family, which are required to maintain duct and pore tube integrity and for the barrier functions of the cuticle and/or eggshell [38]. The similarity of LET-4 and EGG-6 to mammalian small leucine-rich proteoglycans (SLRPs), which bind collagen [41], combined with phenotypes of the mutants, suggests that these proteins are components or regulators of the pre-cuticular and cuticular aECM, and that this aECM is critical for duct/pore integrity. However, other luminal aECM components in these tubes are largely unknown.

One proposed component of the excretory canal tube aECM is the ZP domain and mucin-like protein LET-653 [37,42]. Here we show that LET-653 instead is a component of a transient aECM that precedes cuticle secretion and is essential for excretory duct and pore tube integrity.

## Results

### *let-653* is required for normal excretory duct and pore morphology

**Fig 1:**
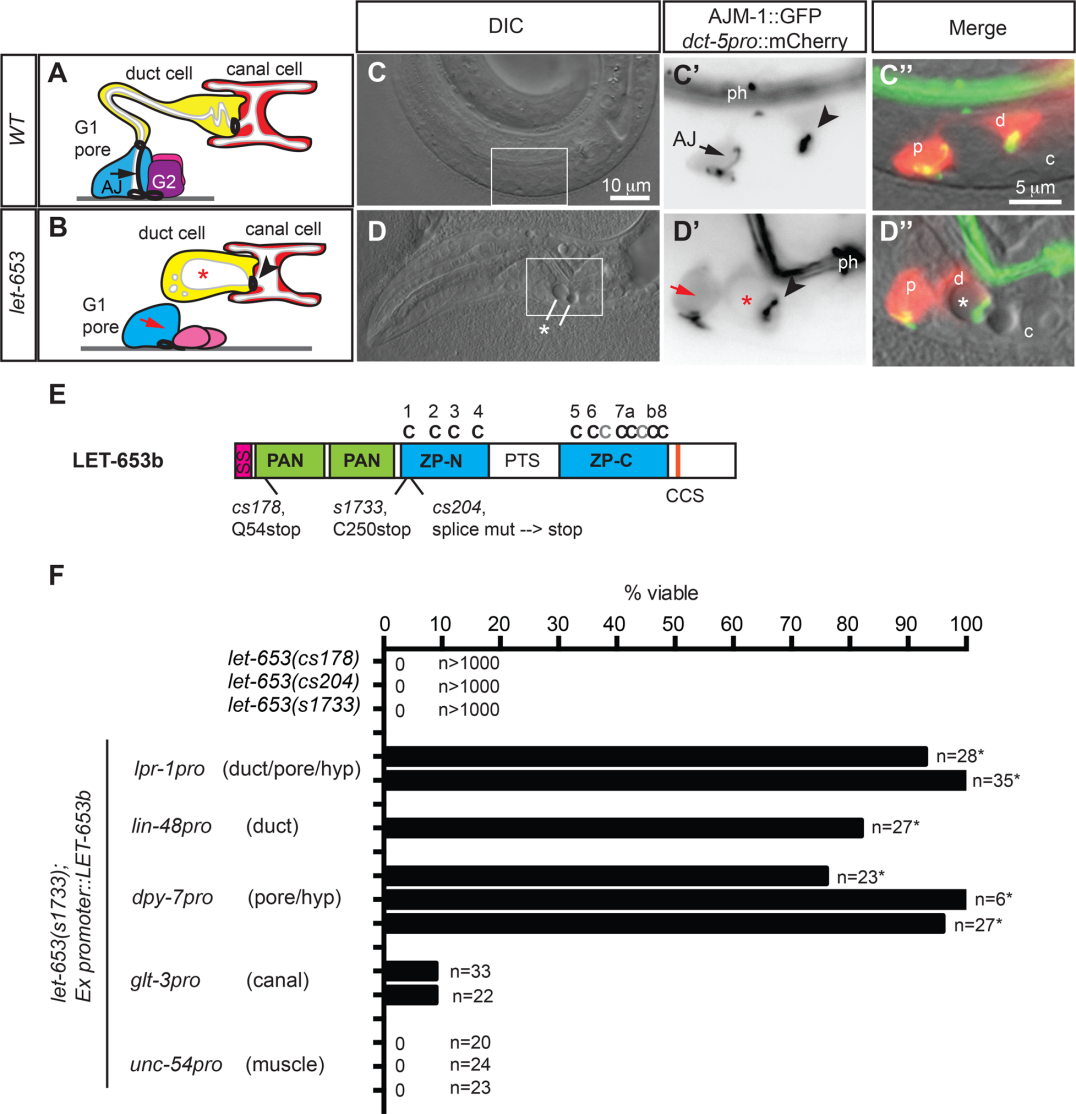
*let-653* acts in the excretory duct and pore cells, not the canal cell. (A-D) *let-653* mutants have morphological defects in the duct and pore. (A,B) Schematics of excretory system morphology in *WT* (A) and *let-653* mutants (B) at the early L1 stage. G1 pore cell is shown in blue, duct cell in yellow, canal cell in red, and G2 and W epidermal cells in pink. White regions represent lumens. Heavy black lines represent apical junctions. Black arrow, pore cell autocellular junction (AJ); red arrow, missing AJ in *let-653* mutant. Arrowheads, duct-canal intercellular junction (IJ), *, duct lumen dilation. Anterior is to the left and ventral is down in all images. (C, D) Duct (d) and pore (p) morphology in WT and *let-653(cs178)* mutants. Cell bodies (red in C”, D”) and all apical junctions (green in C”, D”) are marked as labeled. DIC, differential interference contrast of head; box shows region magnified in subsequent panels. c, canal cell nucleus. ph, pharynx. (E) LET-653b protein schematic and locations of mutant allele lesions. SP, signal peptide. PAN, PAN-Apple domains. ZP, zona pellucida domain. Conserved ZP domain cysteines are indicated above in black; additional cysteines are indicted in grey. PTS, Proline/Threonine/Serine-rich region is predicted by Net-O-Glyc [99] to be highly O-glycosylated. LET-653b is the shortest of three protein isoforms produced from the *let-653* locus by alternative splicing; the other isoforms contain a larger central PTS domain (www.wormbase.org). CCS, consensus furin cleavage site (R-X-R/K-R) [100]. (F) Tissue-specific expression of *let-653b* cDNA in the duct and/or pore efficiently rescued *let-653(s1733)* lethality. Data from multiple independent transgenic lines per construct are shown. *, p<0.01, Fisher’s Exact test, compared to non-transgenic siblings.

The *C*. *elegans* excretory system is a simple, renal-like organ that consists of three tandemly-connected unicellular tubes: the larger excretory canal cell and the smaller duct and pore cells that connect the canal cell to the outside environment for excretion [39,43] (Fig 1A). The canal and duct are seamless tubes, whereas the pore tube is sealed by an autocellular junction (AJ). The three tube cells also connect to each other and the external epidermis via ring-shaped intercellular junctions.

The excretory system is essential for osmoregulation [44], and fluid appears to flow from the canal cell through the duct and pore for excretion [43]. Luminal discontinuity within the pore or duct tubes leads to fluid retention and a characteristic “rod-like” larval lethal phenotype that first manifests as a dilation within the upstream duct or canal lumen [38,45,46] (Fig 1B). To find additional genes important for tube development or maintenance, we conducted an EMS mutagenesis screen for recessive, rod-like lethal mutants with this phenotype, using a strain carrying fluorescent markers for apical junctions (AJM-1::GFP) and for the duct and pore cell bodies (*dct-5pro*::mCherry) in order to visualize duct and pore morphology (Fig 1C and 1D, Materials and Methods). This screen identified 85 lethal mutants with excretory abnormalities, including two new alleles of *let-653* (Fig 1B, 1D and 1E), a gene that had previously been proposed to encode a component of the canal cell glycocalyx [37,42].

*let-653* encodes several protein isoforms that each contain a signal peptide, two PAN-Apple domains, and a C-terminal ZP domain. PAN-Apple domains are putative protein or carbohydrate interaction domains found in plasminogen and clotting factor XI, as well as in various invertebrate cuticle proteins [47,48]. ZP domains are polymerization-competent domains found in many apical matrix proteins, including invertebrate cuticle proteins [3,48–50]. The LET-653 ZP domain consists of a ZP-N subdomain with the typical four cysteines, and a ZP-C subdomain with eight cysteines, including six that are conserved among many ZP proteins and two that are unique to LET-653 (Fig 1E, S1 Fig). A large linker region between ZP-N and ZP-C varies in size and sequence between LET-653 splice isoforms and resembles mucins in that it is rich in proline, serine and threonine residues and predicted to be highly O-glycosylated [42] (Fig 1E, S1 Fig). LET-653 is also N-glycosylated at multiple sites [51] (S1 Fig). LET-653 does not have a transmembrane domain or GPI anchor as assessed by SMART, TMpred and big-Pi [52,53], but it does have a consensus cleavage site (CCS) C-terminal to the ZP domain, similar to other membrane-anchored ZP proteins (Fig 1E). Cleavage at a CCS is thought to be a prerequisite for ZP polymerization and fibril formation [54–57]. In summary, LET-653 contains multiple different domains that are typical of apical ECM factors in all animals.

*let-653*(*cs178)* is a nonsense mutation at codon 54 that is predicted to truncate all LET-653 proteins within the first PAN-Apple domain (Fig 1E); this allele is likely to be null. *let-653(cs204)* is a splice donor mutation, and the original reference allele *s1733* is a nonsense mutation at codon 250; these are predicted to truncate LET-653 proteins near the beginning of the ZP domain (Fig 1E). All three *let-653* alleles are recessive and cause 100% penetrant larval lethality (Fig 1F). This lethality could be rescued by cDNAs corresponding to the shortest *let-653* isoform, *let-653b* (Fig 1F); therefore we used that isoform throughout this study. Mutants for each of the three *let-653* alleles have very similar excretory phenotypes, with luminal dilation occurring in both the excretory canal cell and duct, variable absence of the pore AJ, and detachment of the duct and pore cells (Fig 1D; see below), showing that LET-653 is essential for shaping all three tubes in the excretory system.

### *let-653* acts in the excretory duct and pore cells

*let-653* reporter analyses and tissue-specific rescue experiments indicated that *let-653* functions within the excretory duct and pore rather than in the canal cell (Fig 1F, S2 Fig). A *let-653* promoter::GFP transcriptional reporter was expressed in external (cuticle-producing) epithelial cells, including the epidermis, vulva, rectum and excretory duct and pore, but was mostly excluded from internal epithelia such as the pharynx, intestine and excretory canal cell (S2 Fig). We did occasionally observe *let-653pro*::GFP expression in the canal cells of embryos, but this expression disappeared in later stages (S2 Fig) and we never observed LET-653 fusion proteins in the canal cell (see below). The lethality and excretory defects of *let-653* mutants were efficiently rescued by *let-653* transgenes expressed in the excretory duct (*lin-48* promoter), the excretory pore (*dpy-7* promoter) or both cells (*lpr-1* promoter), but very inefficiently or not at all rescued by transgenes expressed in the excretory canal cell (*glt-3* promoter) or body muscle (*unc-54* promoter) (Fig 1F). Weak rescue using the canal promoter transgene could be attributed to movement of LET-653 protein from the canal cell into the duct and pore (S2 Fig, see below). We conclude that *let-653* acts locally within the excretory duct and pore, and that the canal cell defects described previously [37,42] are a secondary consequence of duct and pore defects.

### *let-653* is required to maintain duct lumen integrity during morphogenesis

**Fig 2:**
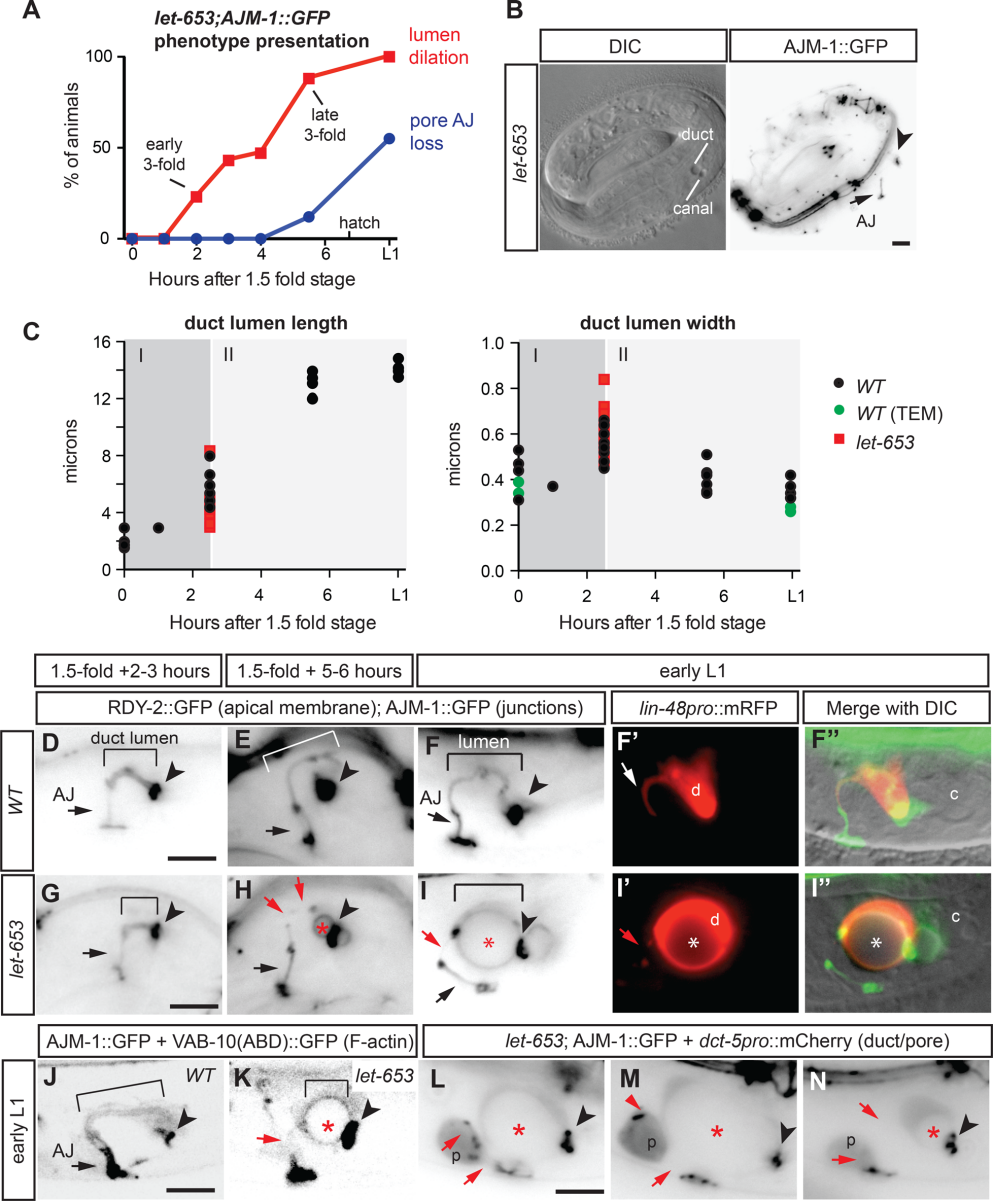
*let-653* is required to maintain lumen integrity during duct tube elongation and narrowing. (A) Time course of duct lumen dilation and G1 pore AJ disappearance in *let-653(cs178)* mutants. Lumen dilation was visualized by DIC as a small bubble at least 1 μm in diameter adjacent to the canal nucleus, and the AJ was visualized with AJM-1::GFP as in panel B. n≥30 for each timepoint. (B) Lumen dilation initiates near the duct-canal junction. DIC and inverted grayscale AJM-1::GFP images of a *let-653(cs178)* 3-fold embryo at early stage of lumen dilation. Note presence of pore AJ (arrow) and duct-canal junction (arrowhead). (C) Duct lumen measurements reveal two distinct phases of lumen growth–elongation and widening (I), followed by elongation with narrowing (II). Duct lumen length and width measurements in WT (black or green) and *let-653(cs178)* mutants (red). Each dot represents a single animal containing RDY-2::GFP and AJM-1::GFP markers as in D-I, except 1.5-fold measurements based on LET-653::SfGFP marker, and green dots based on *WT* TEM specimens. For *let-653*, only young 3-fold embryos with an intact duct lumen were scored. The appearance of lumen fragmentation and dilation prevented accurate measurements in older animals. (D-I) In *let-653* mutants, the distal duct lumen fragments during elongation and narrowing. Bracket indicates duct lumen. Black arrow indicates pore AJ. Arrowhead indicates duct-canal junction. Red arrows point to lumen or junction discontinuities. Asterisks indicate duct lumen dilation. Inverted grayscale fluorescent images of staged WT (D-F) and *let-653(cs178)* (G-I) animals expressing the apical membrane marker RDY-2::GFP and junction marker AJM-1::GFP. RDY-2::GFP also marks the canal lumen, which shows up prominently near the duct-canal junction in some images. F and I also include the cytoplasmic duct marker *lin-48pro*::mRFP. (J-N) The pore loses its AJ and intercellular junctions in *let-653(cs178)* L1 larvae. (J,K) F-actin visualization using confocal microscopy of VAB-10(ABD)::GFP. F-actin localizes near apical membranes and junctions in both WT (J) and *let-653* mutants (K). (L-N) Pore AJ loss and duct-pore separation after hatch. Inverted grayscale images of *let-653* mutants expressing the cytoplasmic marker *dct-5pro*::mCherry and junction marker AJM-1::GFP. Scale bars, 5 μm.

To better understand the requirements for *let-653* in the excretory duct and pore, we examined apical junctions (marked with AJM-1::GFP) and the apical membrane (marked with RDY-2::GFP; [58]) or apical actin (marked with *dct-5pro*::VAB-10ABD::GFP; [59]) in staged embryos and larvae (Fig 2). *let-653* mutants appeared normal through the early 3-fold stage (Fig 2A and 2G), indicating that the duct and pore had successfully wrapped to form polarized tubes and that *let-653* is not required to establish cell junctions or to form an initial lumen. Lumen dilations first appeared in the duct between the early-and mid 3-fold stages (Fig 2A–2C), several hours before the bulk of cuticle secretion [40]. Much later, around the time of hatching (and after duct dilations had become quite severe), the pore AJ disappeared and the pore detached from either the duct or its ventral epidermal partner G2, while remaining attached to the other (Fig 2A and 2K–2N).

We focused our analyses on the duct, since mutant defects first appeared in this cell. Examination of wild-type embryos revealed two phases of duct lumen morphogenesis (Fig 2A and 2D–2F). During phase I, between the 1.5-fold and early 3-fold stages, the duct lumen increased in both length and diameter. During phase II, between the early and late 3-fold stages, the duct lumen elongated even further, but slightly narrowed in diameter, suggesting apical constriction. By the time of hatch, the duct lumen measured ~14 microns long but was less than 400 nm in diameter.

In *let-653* mutants, the duct lumen did initially elongate and widen to some degree, but lumen diameter appeared increasingly irregular during the phase II of elongation (Fig 2A and 2G–2H). Lumen dilation always initiated near the intercellular junction between the duct and canal cells (Fig 2C and 2H), and coincided with a very thin and/or fragmented appearance of the lumen in the more distal part of the duct (Fig 2H and 2I), where a very narrow cellular process, only slightly wider than the lumen, normally connects the duct to the pore (Fig 2F). By L1, this narrow process often disappeared completely and the duct and pore appeared detached (Fig 2L–2N). The continued presence of RDY-2::GFP and F-actin at the remaining proximal (now dilated) duct apical membrane, as in WT, indicated that cell polarity remained intact (Fig 2D–2K). These observations suggested that lumen dilations occur as a result of fluid accumulation due to lumen blockage.

**Fig 3:**
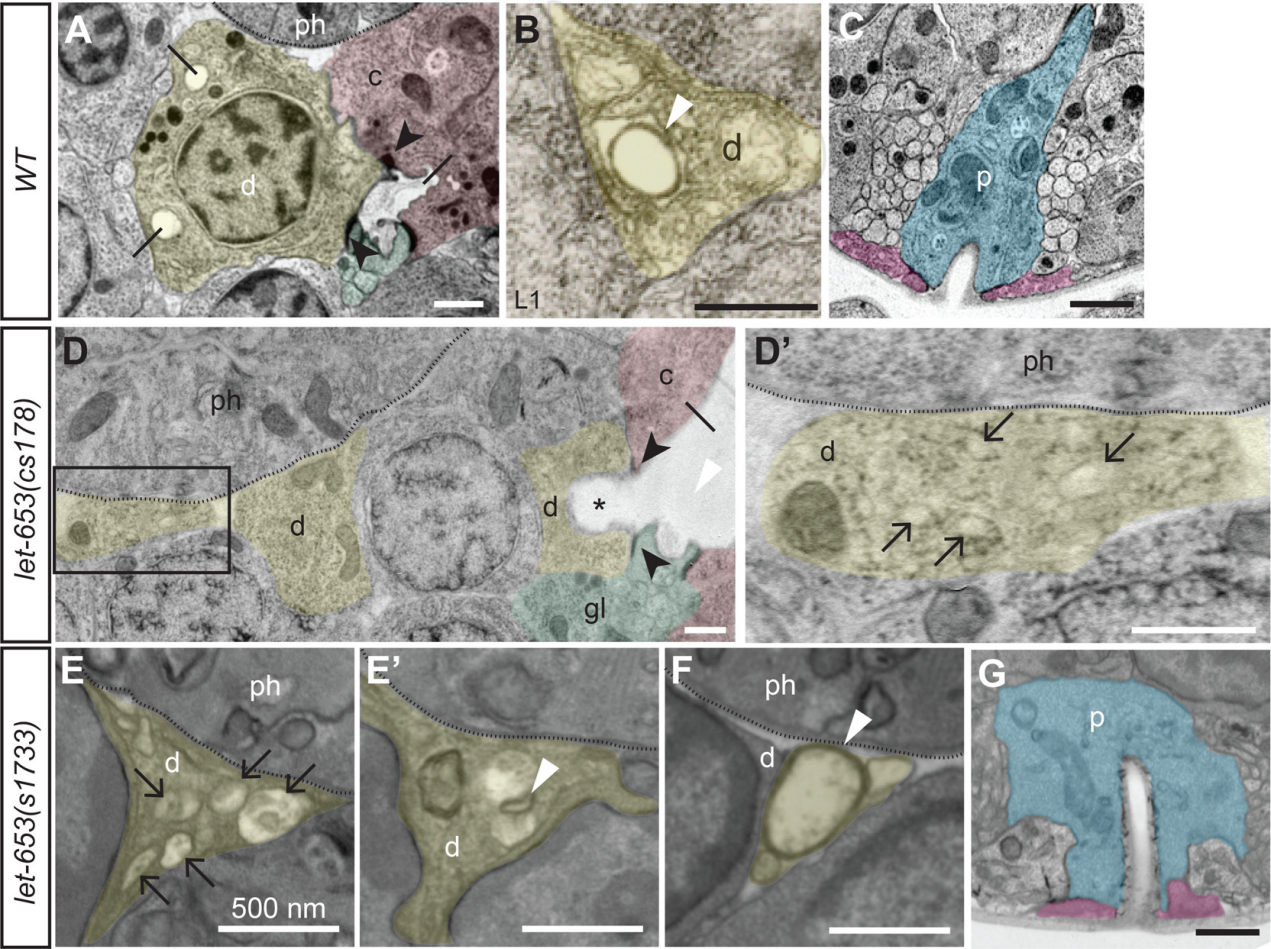
TEM analysis of *let-653* mutant excretory system. TEM sections of the excretory duct or pore region in WT (A-C) or *let-653* mutants (D-G), pseudocolored to indicate the duct (d, yellow), G1 pore (p, blue), G2 (pink), canal cell (c, red) and excretory gland cell (gl, green). Dotted lines indicate basement membrane of the pharynx (ph). (A) Lateral section from a normal, late 3-fold stage embryo, showing the duct cell body and the luminal connection between the duct and canal cells. Black arrowheads indicate intercellular junctions, and lines indicate lumen. Multiple luminal segments appear in a single cross-section due to the winding path of the wild-type duct lumen. (B) Transverse section through the duct process in a normal L1 larva. White arrowhead indicates the cuticle lining of the duct lumen. (C) Excretory pore opening from the same animal as in A. (D, D’) Skewed lateral section through a *let-653(cs178)* late 3-fold stage embryo. A portion of the duct lumen (asterisk) and the canal cell lumen are greatly dilated behind a region devoid of lumen. Most of the duct cell body and the duct nucleus are outside the plane of this section; the central nucleus belongs to a different cell. Boxed region in D is magnified in D’; this region corresponds to the beginning of the duct process (compare to B). This region lacks a continuous lumen, but instead contains multiple smaller membrane-bound structures (black arrows). (E-F) Transverse sections from the duct processes of two *let-653(s1733)* L1 larvae. The lumen is fragmented (E) or contains abnormally small (E’) or large (F) rings of cuticle-like material (compare to B). (G) The G1 pore is intact and has a normal lumen diameter (compare to C) in this mutant L1. Scale bars, 500 nm.

Transmission electron microscopy (TEM) analysis of serial sections in one *let-653(cs178)* 3-fold embryo and two *let-653(s1733)* L1 larvae confirmed duct lumen discontinuities and revealed multiple, small membrane-bound compartments (3/3 animals) instead of a single lumen (Fig 3). In mutant L1s, remaining segments of duct lumen were lined by abnormally small or abnormally large rings of cuticle-like material (Fig 3E and 3F), indicating that cuticle secretion had proceeded after lumen fragmentation and dilation. The pore lumen remained intact in the embryo and one of the L1 mutants, and had a normal diameter (Fig 3C and 3G). The canal lumen was greatly dilated immediately upstream of the duct-canal junction (Fig 3D), but otherwise intact and fairly normal further upstream of this connection point. It therefore appears that the duct tube is particularly sensitive to *let-653* loss, and that duct and canal lumen dilation occurs as a secondary consequence when excretory fluid flowing from the canal cell backs up behind a distal duct luminal discontinuity, as has been suggested for other mutants [38,45]. We conclude that LET-653 is needed to maintain duct apical membrane integrity and a continuous, open lumen during elongation and narrowing.

### *let-653* is required to shape other epithelial apical compartments

**Fig 4:**
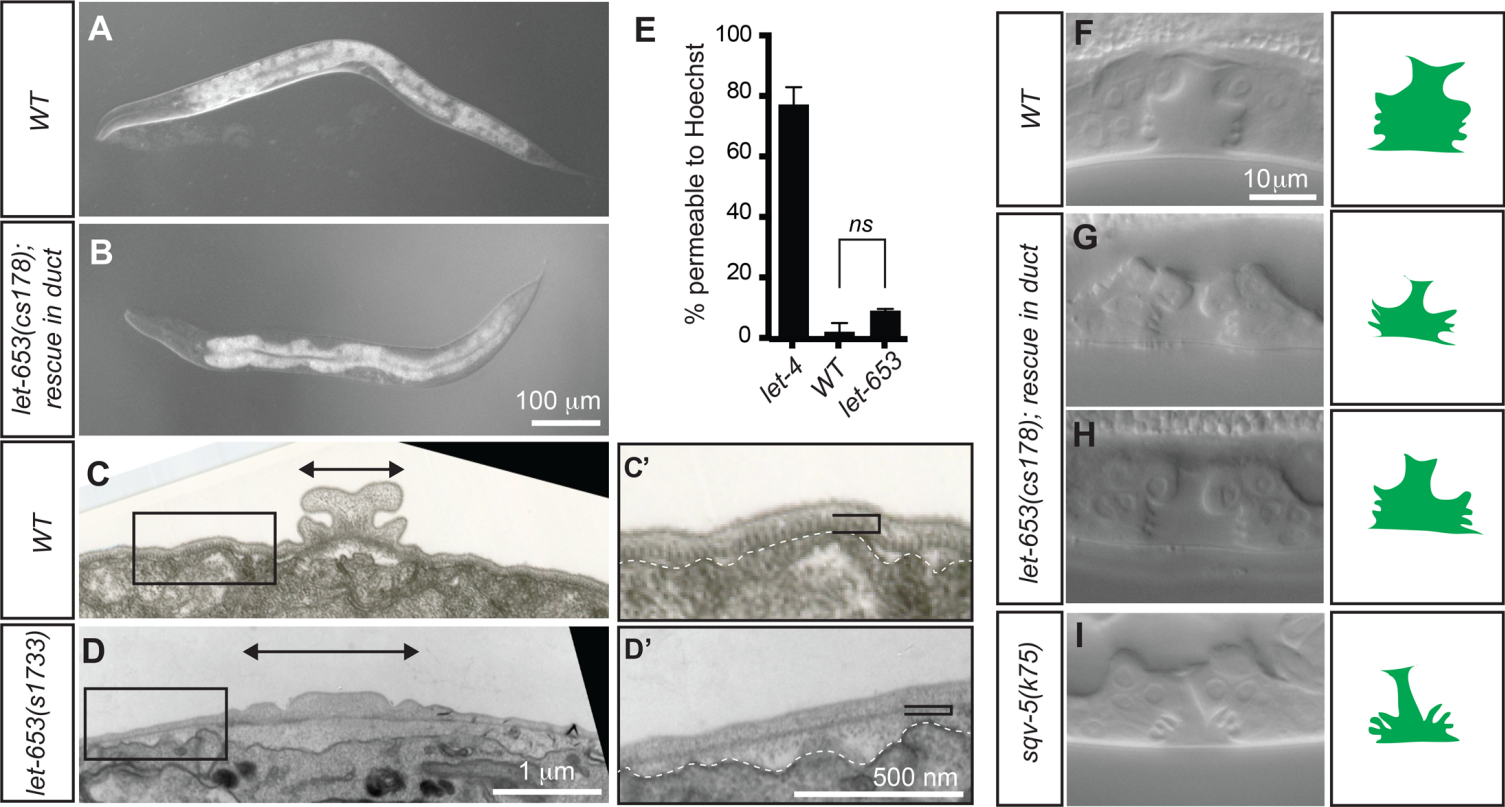
*let-653* is required for proper alae formation and vulva lumen shaping. (A,B) Comparison of body shape in WT and *let-653(cs178)* L4 larvae rescued to viability with a duct-specific *lin-48pro*::LET-653::SfGFP transgene. The mutants are slightly shorter and fatter than WT. (C,D) TEM images of alae in WT and *let-653(s1733)* L1 larvae. Transverse sections through the head region at the posterior bulb of the pharynx are shown. Boxed regions are magnified in adjacent panels. Arrowed bars indicate width of the alae. (C’, D’) *let-653* mutants have alae and cuticle organization defects. White dotted line indicates edge of the epidermal cell layer. In *let-653* mutants, the striated layer of the cuticle (black bracket) appears thin or absent, and the space between this layer and the underlying epidermis is expanded and filled with diffuse, lightly staining material. (E) The permeability barrier is intact in *let-653* mutants. L1 larvae were incubated in 2 ug/ml Hoechst dye in M9 for 15 minutes and then rinsed and scored for nuclear fluorescence. *let-4(mn105)* was used as a positive control for permeability [38]. Data from three replicates shown, n>15 per genotype for each experiment. No significant difference found between *let-653* and WT permeability by Student’s *t-*test, two-tailed. (F-I) Comparison of L4 vulva lumen morphology in WT (F), duct-rescued *let-653* mutants (G,H) and a hypomorphic chondroitin biosynthesis mutant *sqv-5(k175)* [101]. Animals were staged as mid-L4 based on fusion of the anchor cell and horizontal orientation of the ventral vulva “fingers”. Modest vulval expansion defects or asymmetries were observed in 11/31 of mutant larvae.

Consistent with the observation that *let-653* is widely expressed in external epithelia (S2 Fig), *let-653* mutants exhibited additional defects in shaping of the epidermis and vulva. The epidermis and its overlying aECM normally undergo circumferential apical constriction to shape the embryo into an elongated worm (Fig 4A) and to form specialized cuticular ridges, termed alae, that run along its lateral surfaces (Fig 4C) [50,60,61]. When rescued to viability with a heterologous duct promoter construct (Fig 1G, S2 Fig), *let-653* mutants had a moderately short and fat (Dumpy) body morphology (Fig 4B). Furthermore, TEM analysis revealed that the L1 alae of *let-653* mutants were flatter and wider than in *WT* (Fig 4C and 4D). The internal striated layer of the cuticle was present but thinner than normal, and the space between the cuticle and the epidermis was expanded (Fig 4C’ and 4D'). The Dumpy and flat alae defects are similar to (but less severe than) those reported for mutants in ZP domain cuticulins [50], and suggest a defect in apical constriction of the lateral epidermis, possibly due to failure to appropriately connect the epidermis to the cuticle. Despite these defects in body shaping and cuticle morphology, the barrier function of the cuticle remained largely intact in *let-653* L1 larvae (Fig 4C).

Expression of *let-653* was also observed in the vulva (S2 Fig), which is a large multi-cellular tube used for egg-laying. Shaping of the vulva lumen requires both an expansive force generated by chondroitin proteoglycans [19,62], and a constrictive force generated by circumferential actomyosin contraction [63]. Duct-rescued *let-653* mutants exhibited variable abnormalities in the shape of the vulval lumen, including asymmetries and general under-expansion along the dorsal-ventral axis (Fig 4F–4H). These defects were incompletely penetrant and much milder than those reported for either chondroitin biosynthesis mutants such as *sqv-5* (Fig 4I) [62] or Rho kinase mutants [63], but suggest a role for LET-653 in expanding vulval lumen dimensions.

The non-transgenic progeny of duct-rescued *let-653* animals arrested as L1 larvae with the typical *let-653* excretory phenotype. Therefore, *let-653* is not required for other chondroitin- or aECM-dependent processes such as eggshell formation [64], cytokinesis [65], or pharyngeal-epidermal attachment [66].

### LET-653 localizes transiently to two distinct pre-cuticular apical ECM compartments

**Fig 5:**
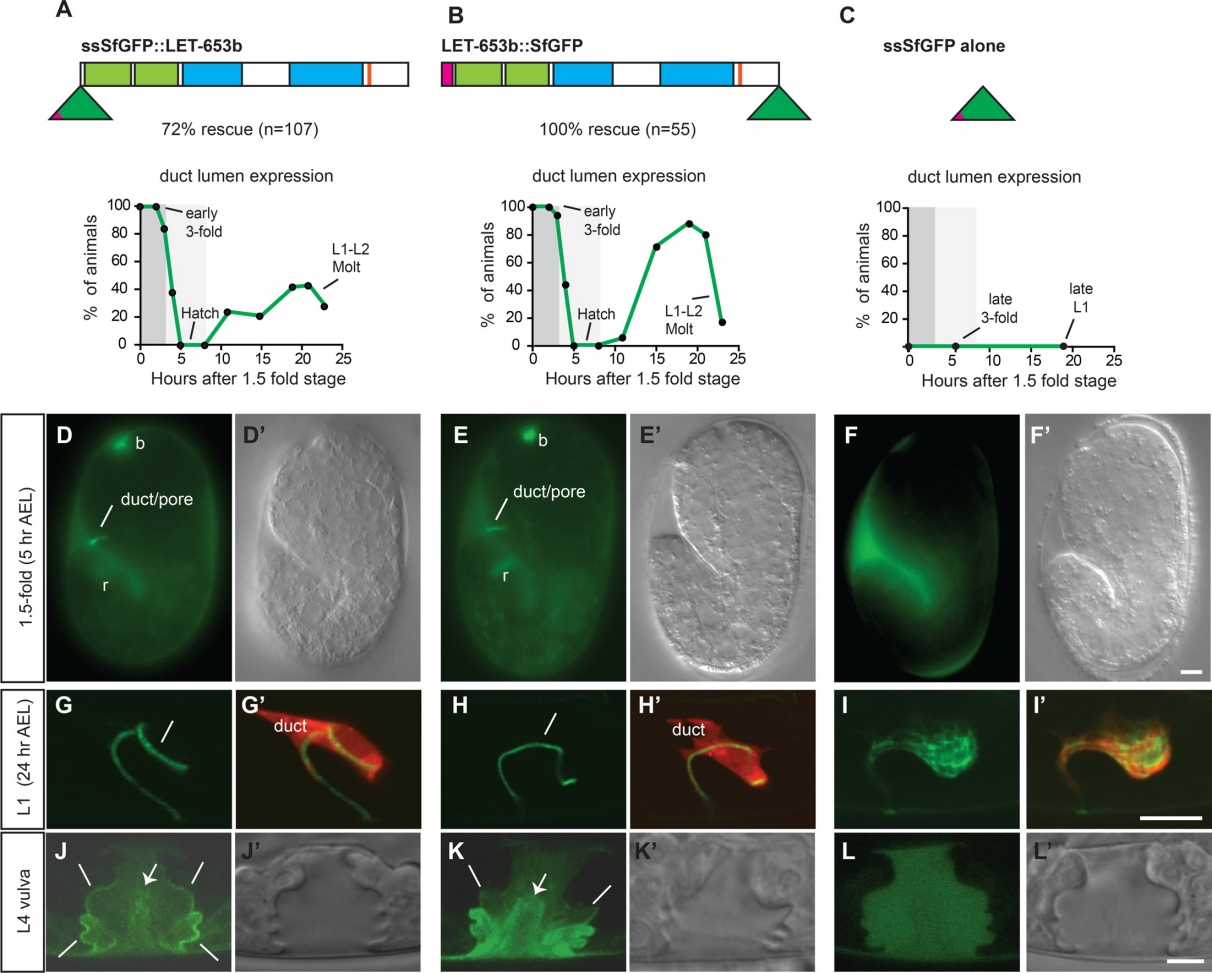
LET-653 is a component of a transient pre-cuticular apical ECM. (A-B) Translational fusions of LET-653 to SfGFP rescue *let-653(cs178)* mutant lethality and show a cyclic pattern of accumulation in the duct lumen that precedes cuticle deposition. Expression data are from fusions visualized in an otherwise wild-type background. Protein domains are schematized as in Fig 1E. Green triangle represents SfGFP and pink triangle represents signal sequence (ss). n≥15 animals for each timepoint. AEL, after egg lay. Grey shading indicates phases I and II of duct lumen elongation as in Fig 2C. (C) ssSfGFP alone does not accumulate in the duct lumen. (D-F) At 1.5-fold, LET-653 fusions accumulate between the embryo and the eggshell, and in duct (lines), buccal (‘b’) and rectal luminal (‘r’) regions, while ssSfGFP accumulates only between the embryo and eggshell. Epifluorescent and corresponding DIC images are shown. (G-I) LET-653 fusions, but not ssSfGFP alone, re-accumulate in the late L1 larval duct lumen. Confocal slices. (G’,H’,I’) *lin-48pro*::mRFP marks the duct cell. (J-L) In the L4 vulva lumen, LET-653 accumulates near the apical membrane (lines) and along fibrils within the luminal core (arrow). ssSfGFP alone has no such preferential localization. Confocal slices. D’,E’,F’,J’,K’,L’ show DIC images for comparison. (C-L) WT background. Scale bars, 5 μm.

To visualize the LET-653 protein, we used the *let-653* promoter to express LET-653 either N- or C-terminally tagged with Superfolder (Sf) GFP, which has been reported to fold stably in oxidizing extracellular environments [67]. Both fusions rescued *let-653* mutant lethality (Fig 5A and 5B), indicating that the tagged proteins are functional.

**Fig 6:**
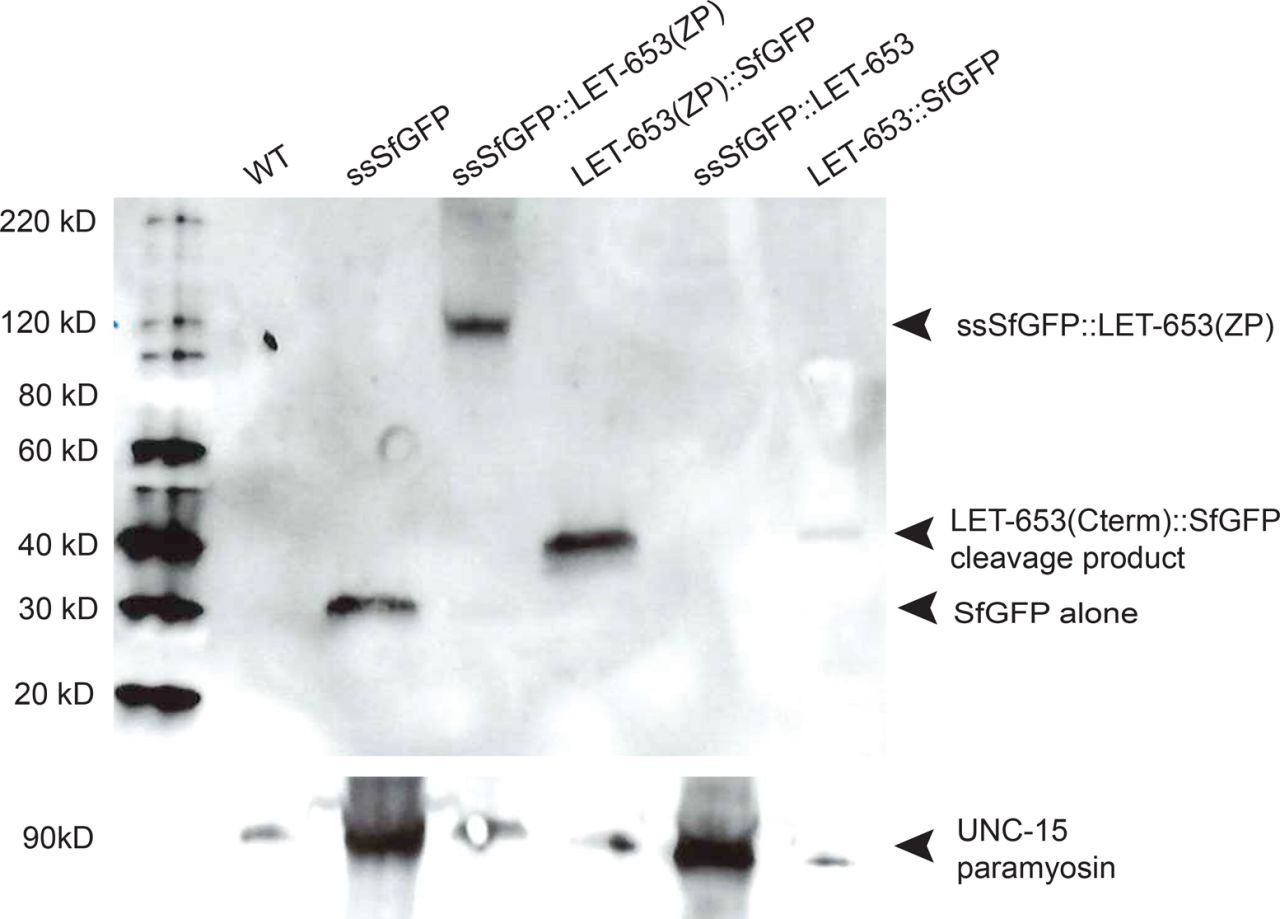
LET-653 is cleaved at its C-terminus. Anti-GFP Western blot performed on 1.5-fold transgenic embryos. Full-length LET-653 fusions were expected to be found at 100kD, or higher if post-translationally modified. These were not detected, but fusions lacking the PAN domains were readily observed. C-terminally tagged LET-653 yields a band just below 40kD (arrowhead), indicating that SfGFP and about 80 amino acids of LET-653’s C-terminus have been cleaved from the full-length protein, consistent with cleavage at the predicted CCS. The loading control antigen is UNC-15/paramyosin. Blot shown is representative of n = 11.

To test if LET-653 is cleaved as described for other ZP proteins, we examined the fusion proteins via Western blotting. Western analyses of lysates from transgenic embryos failed to detect the full-length fusion proteins, but did consistently detect ZP domain fusions lacking the PAN domains as well as release of the C-terminal, but not the N-terminal, SfGFP tags (Fig 6). These data confirm that LET-653 can be cleaved, likely at the CCS. Furthermore, as expected if significantly O-glycosylated, the N-terminally tagged LET-653(ZP) fusion ran at ~120kD despite a predicted molecular weight of only ~80kD. Surprisingly, both N- and C-terminally tagged LET-653 fusions showed very similar patterns of localization *in vivo* that were different from the localization of secreted (ss) SfGFP alone (Fig 5). Therefore, either a significant proportion of LET-653 must remain uncleaved, or the cleaved portion must remain non-covalently associated with the mature form. The latter model is consistent with *in vitro* studies of mammalian ZP3, which showed very slow dissociation of the C-terminal region after ZP3 cleavage [68].

LET-653 translational fusions were apically secreted and accumulated transiently in cycles that preceded cuticle deposition (Fig 5). Beginning at ventral enclosure, LET-653 fusions accumulated throughout the region between the embryo and the inner layer of the eggshell (S2 Fig), indicating apical secretion prior to and during formation of the embryonic sheath, the first known layer of the cuticle [60,69]. By the 1.5-fold stage, LET-653 fusions (but not ssSfGFP alone) also accumulated within the newly-formed lumen of the excretory duct and pore (Fig 5D and 5E). LET-653 remained extra-embryonic and luminal for the next 3–4 hours, but then disappeared prior to the bulk of cuticle secretion (Fig 5A and 5B). This temporal pattern indicates that LET-653 is present throughout phase I of duct lumen growth, but disappears during phase II, as the lumen narrows. Therefore, LET-653 may act during the first phase of lumen growth to protect against lumen fragmentation in the second phase.

LET-653 reappeared transiently within the duct and pore lumen in the latter part of each subsequent larval stage (Fig 5A, 5B, 5G and 5H). It did not detectably incorporate into the body cuticle, but accumulated in the space between the new and old cuticles during molting (S2 Fig), and within the vulval luminal space during the mid-L4 period when the epidermal cuticle separates that luminal space from the outside environment (Fig 5J and 5K). In the vulva, where resolution was highest due to the large size of the luminal cavity, both LET-653 fusions were enriched near apical membranes and also present in wispy fibrils within the central lumen cavity; these fibrils extended along the ventral border of the lumen to contact the apical membrane of the vulB cells (Fig 5J and 5K). ssSfGFP alone appeared uniformly distributed (Fig 5L), indicating that the LET-653 pattern is specific. We conclude that LET-653 is part of an early and transient pre-cuticular aECM.

**Fig 7:**
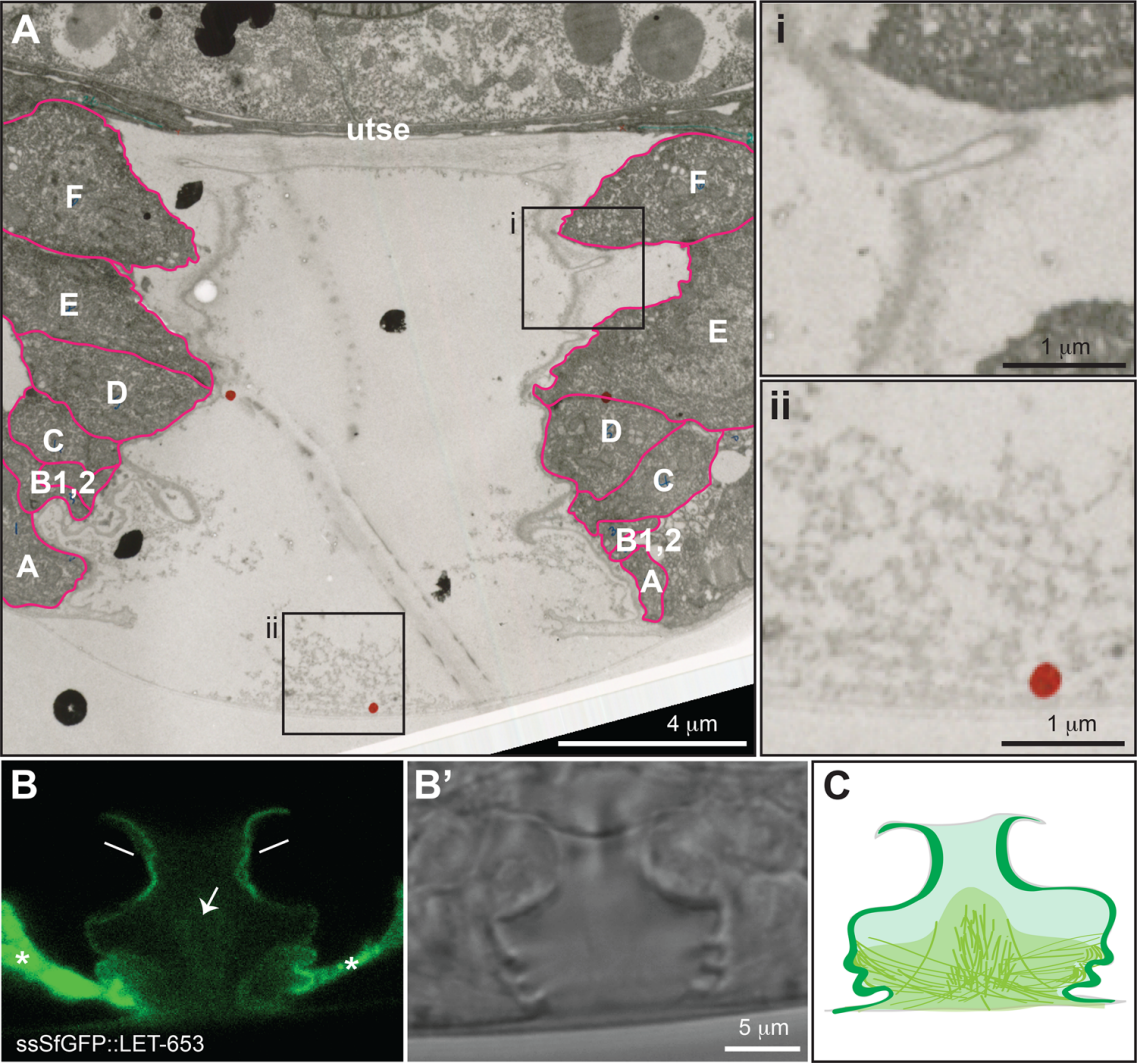
TEM visualization of the vulval aECM. A) TEM image of a wild-type mid-L4 vulva, showing two discrete aECM compartments. Boxed regions are magnified in panels i and ii. Red dot in panel ii is an artifact of original annotation. Fuschia lines outline cells in the vulA-F rings. B, B’) Confocal slices focused to the dorsal vulva, showing ssSfGFP::LET-653 localization near the apical membrane. Asterisks indicate intracellular accumulation of the fusion protein. C) Schematic representation of LET-653 localization to distinct luminal matrix pools.

We compared the LET-653 localization pattern in the vulva to archival TEM images of the vulva lumen (Fig 7A). TEM reveals a prominent matrix layer that lines the luminal membrane; this layer is thickest at the dorsal apex of the vulva, near the vulE and vulF cells, but continuous along the entire luminal membrane, and has a discrete, darkly staining outer border. A loosely organized fibrillar matrix, which is variably preserved in different sections, fills the ventral portion of the lumen and abuts the apical matrix near the vulA-C cells. The LET-653 localization pattern closely matches these two matrix compartments observed by TEM (Fig 7B and 7C). The excretory duct and pore tubes are too narrow to resolve different matrix compartments by confocal imaging, but our previous TEM data are consistent with a similar two-compartment organization of the pre-cuticular aECM in those tubes [38]. In summary, in the vulva and likely in the excretory duct and pore tubes, LET-653 localizes transiently to two distinct pre-cuticular aECM compartments.

### Post-transcriptional mechanisms regulate LET-653 cyclic luminal retention

The oscillatory pattern of LET-653 accumulation in the duct and pore lumen may result in part from transcriptional regulation, since published RNAseq data show that *let-653* transcripts oscillate with the molt cycle during larval development, with peak expression in the inter-molt period prior to cuticle collagen expression [70]. However, LET-653::SfGFP driven by the constitutively active *lin-48* (duct) promoter also showed an oscillatory pattern of accumulation within the duct and pore lumen (S2 Fig), showing that post-transcriptional regulation also occurs. Furthermore, LET-653::SfGFP driven by the constitutively active *glt-3* (canal) promoter accumulated within the canal lumen (and sometimes within the duct and pore lumen; S2 Fig) only during embryogenesis, and was rarely if ever detected in larvae, possibly explaining the minimal rescue activity seen with *glt-3* promoter transgenes (Fig 1G). Finally, secreted ssSfGFP alone, when driven by the *let-653* promoter, never accumulated to detectable levels in the duct and pore lumen (Fig 5C, 5F and 5I). These observations suggest that LET-653 luminal retention depends upon the correct site of synthesis and requires interactions between LET-653 and some anchoring factor(s).

### The LET-653 PAN and ZP domains mediate distinct interactions at the luminal core and apical membrane

**Fig 8:**
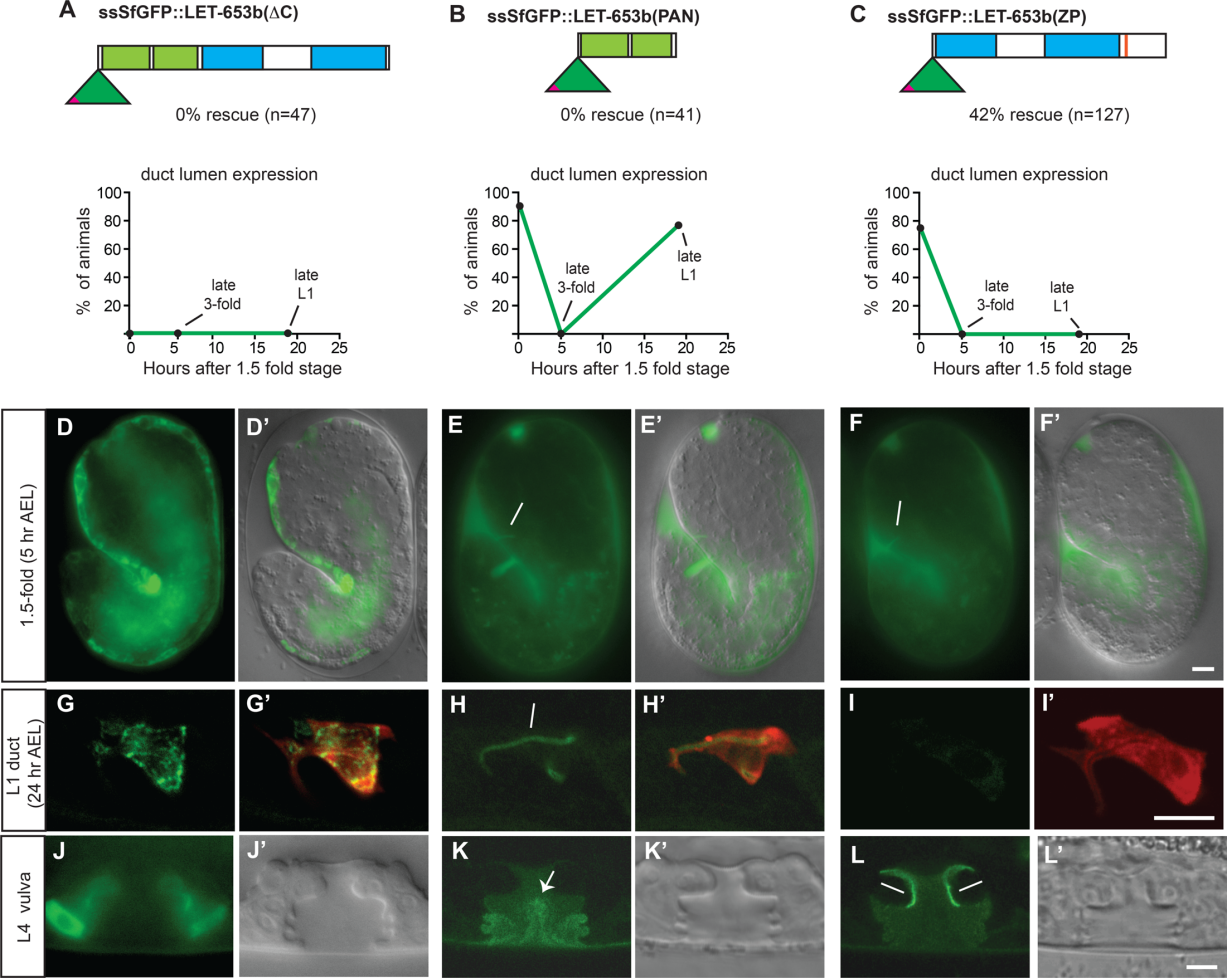
LET-653 PAN and ZP domains confer distinct localization patterns and mediate duct lumen cycling and protection, respectively. (A-C) LET-653 fusions lacking the C-terminal, ZP or PAN domains showed differential functions and localization in the duct. Only LET-653(ZP) rescued mutant defects. (D,G,J) LET-653(∆C) accumulated intracellularly rather than extracellularly, suggesting a failure in secretion. (E-F) LET-653(PAN) and LET-653(ZP) fusions localized normally to the duct in 1.5 fold embryos. (H-I) LET-653(PAN) accumulated in the late L1 larval duct lumen, but LET-653(ZP) did not. Confocal slices. (G’,H’,I,) *lin-48pro*::mRFP marks the duct cell. (K) LET-653(PAN) associated with fibrous material in the center of the lumen (arrow) (L) LET-653(ZP) associated with the dorsal apical membrane region. D’,E’,J’,K’,L’ show DIC images for comparison. Scale bars, 5 μm.

To identify domains of LET-653 important for function, localization and cycling, we analyzed N-terminally tagged fusion proteins lacking the PAN, ZP or C-terminal domains (Fig 8). A LET-653 variant truncated at the CCS (LET-653∆C) was non-functional and retained within cells (Fig 8A, 8D, 8G and 8J), consistent with prior studies showing that the proper trafficking and apical secretion of ZP proteins requires hydrophobic sequences C-terminal to the cleavage site [55,57], which form an integral part of the non-polymerized ZP-C fold [56,68]. Indeed, shorter variants lacking the entire ZP-C subdomain were normally secreted (Fig 8B, 8E, 8H and 8K, S3 Fig), indicating that the C-terminus is essential for secretion only in the presence of the ZP-C domain. These truncated LET-653 constructs were non-functional in *let-653(cs178)* rescue assays (Fig 8B and S3 Fig). In contrast, a LET-653 variant lacking the PAN domains (LET-653(ZP)) was still partly functional (Fig 8C). We conclude that the ZP domain is critical for function, but the PAN domains are at least partly dispensable.

LET-653(ZP) and LET-653(PAN) fusions showed distinct patterns of spatial and temporal localization. LET-653(ZP) accumulated in the duct lumen of embryos and then was downregulated normally, but it was not detectably retained within the duct lumen of L1 larvae (Fig 8C, 8F and 8I). In the vulva, LET-653(ZP) specifically associated with the dorsal portion of the apical membrane (Fig 8L), a small subset of the locales normally occupied by the full-length protein (Figs 5J, 5K and 7B). Conversely, LET-653(PAN) showed a normal cyclic pattern of accumulation in the duct lumen of both embryos and larvae (Fig 8B, 8E and 8H), and labeled vulval luminal fibrils but was not enriched near apical membranes (Fig 8K). Similar localization was observed for constructs also retaining the mucin and/or ZP-N domains (S3 Fig), indicating that the C-terminal portion of the ZP domain is required for membrane-proximal localization.

We draw several conclusions from these data. First, the main role of the PAN domains is to help anchor the functional ZP domain in the correct location, perhaps preventing its flow-induced displacement. This role is less critical in the embryonic duct, where flow is presumably weaker, but is both necessary and sufficient for cyclic duct lumen retention in larvae. Second, the PAN and ZP domains interact with different binding partners in different locations–the PAN domain binds to a partner in the luminal core, while the ZP domain binds to a partner near the apical membrane. Third, ZP-C-dependent interactions at the apical membrane during tube morphogenesis correlate with LET-653 duct lumen protective activity. Finally, LET-653 reappearance at later stages is apparently dispensable.

### LET-653 has a ZP-dependent lumen expanding activity

**Fig 9:**
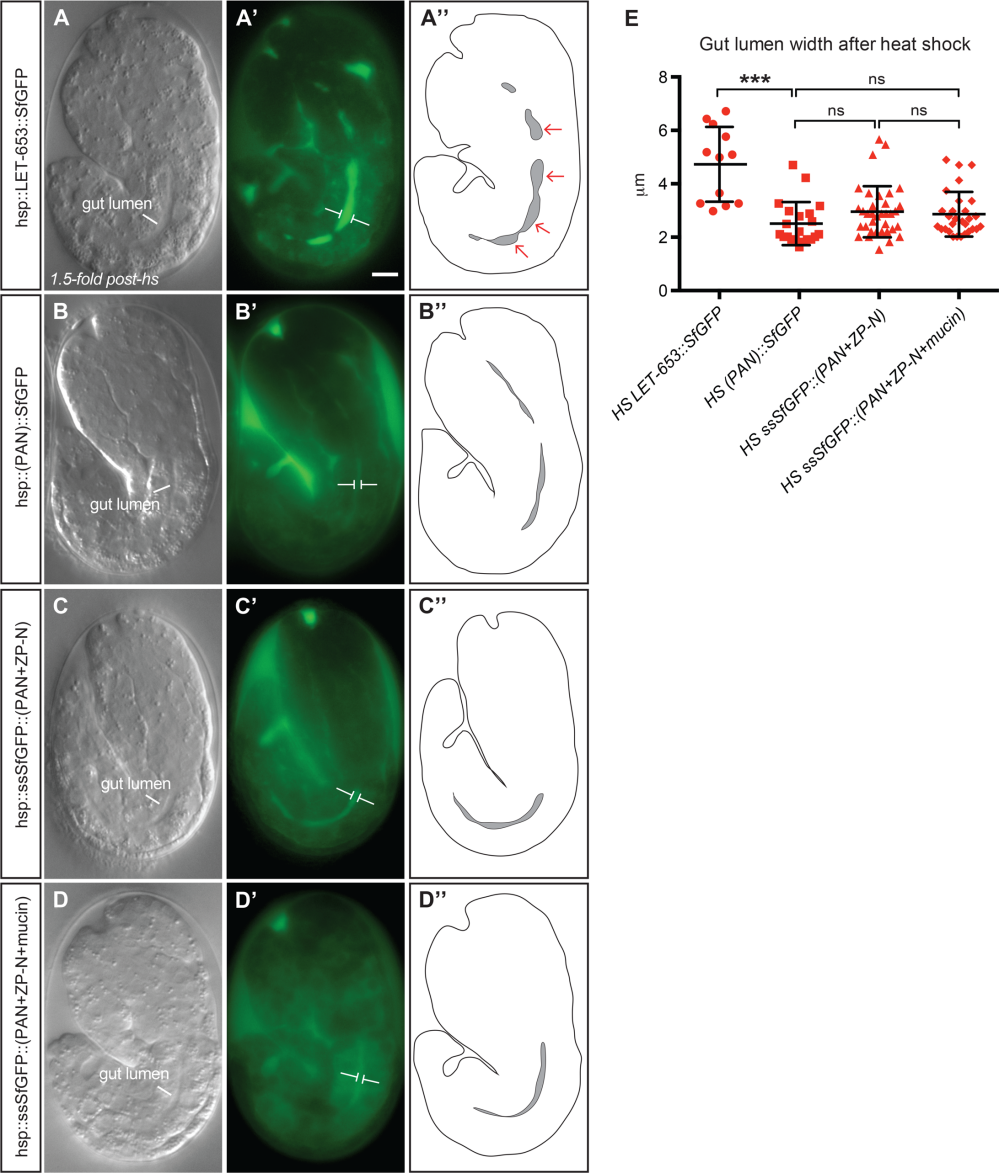
LET-653 is sufficient to expand the gut lumen. (A-D) Representative 1.5-fold embryos after heat shock overexpression of full-length LET-653 (A), LET-653(PAN) (B), LET-653(PAN+ZP-N) (C) or LET-653(PAN+ZP-N+mucin) (D) fusions under the *hsp16*.*41* promoter. Gut lumen expansion is visible by DIC in panel A. (A’-D’) GFP expression. Tagged protein was expressed and secreted to similar degrees for the first three genotypes, but secreted more variably for the final genotype. White brackets flank lumen edges at the widest point along the gut length. Scale bar, 5 μm. (A”-D”) Schematic outlines of the entire embryo and gut (lumen shaded dark gray). Narrow regions alternate with wide “bulbs” (red arrows), suggesting a local expansion effect. (E) Individual plotted measurements of gut lumen width in microns. n = 13 for full-length LET-653 and n≥21 for the others. Median, 25th percentile, and 75th percentile are shown for each. *** p<0.001, Student’s *t*-test, two-tailed.

The above data indicate that LET-653 is present during periods of lumen expansion and is required for proper lumen dimensions. To test if LET-653 is sufficient to expand lumen diameter, we used a heat shock promoter to express LET-653 in the gut tube, where it is not normally present. The gut tube is not cuticle-lined and therefore relevant LET-653 partners also may be absent. Full-length LET-653 could expand gut lumen diameter more than two-fold (which equates to a four-fold increase in tube volume), whereas LET-653(PAN) and the other truncated constructs lacking the ZP-C domain had either no effect or a very modest effect (Fig 9). Lumen expansion was irregular along the gut tube, suggesting localized action rather than a uniform hydrostatic force. We conclude that LET-653 has a ZP-dependent lumen-expanding activity, and this activity cannot be explained solely by the presence of the mucin-like linker or by effects on cuticle organization.

### Stable ZP-dependent interactions at the apical membrane occur independently of C-terminus removal

**Fig 10:**
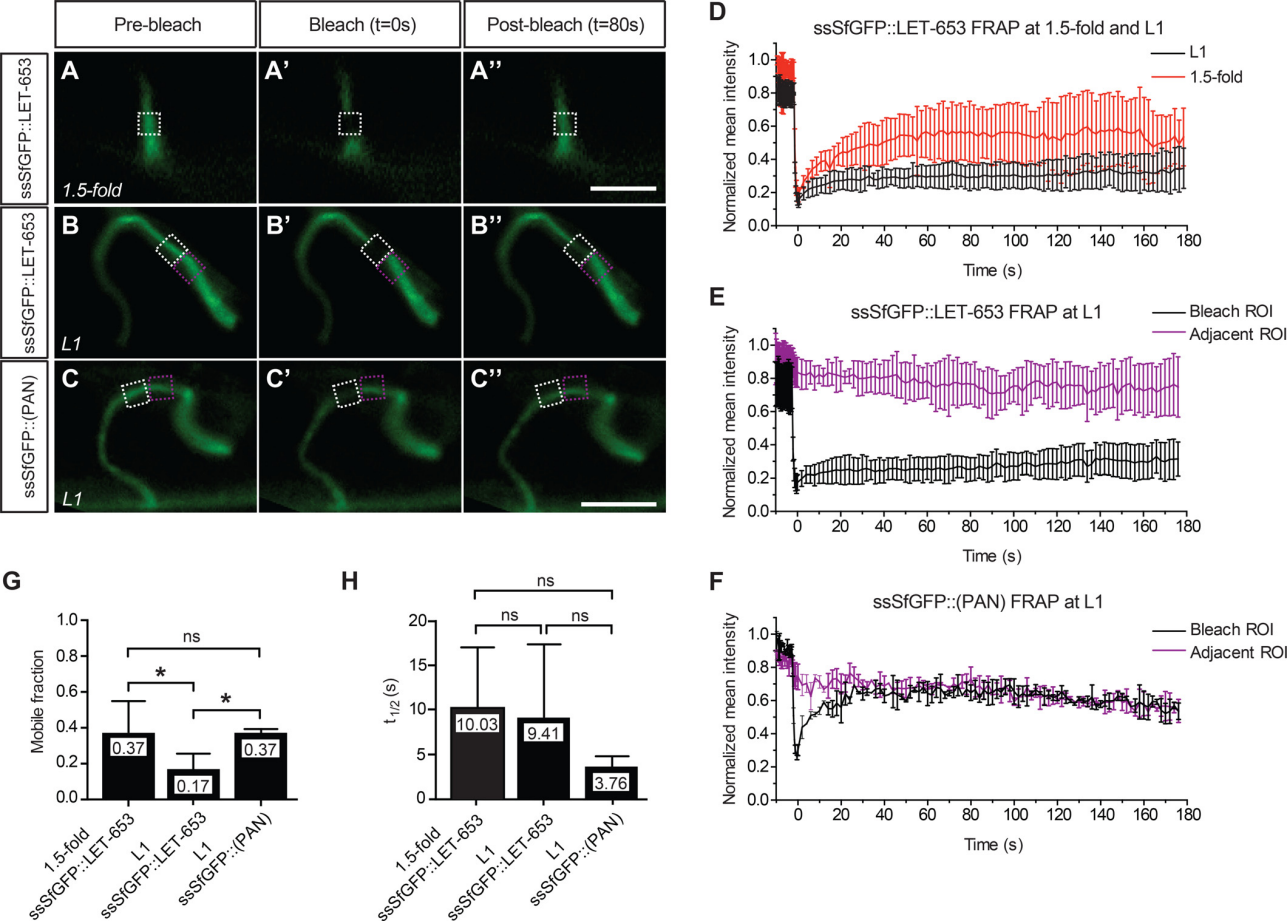
Dynamic PAN- and stable ZP-mediated interactions in the duct lumen. Fluorescence recovery after photobleaching (FRAP) of LET-653 translational fusions in the duct lumen. (A-C) Pre-bleach, bleach, and post-bleach frames taken from FRAP experiments on the indicated fusions in 1.5-fold embryos (A-A”) or L1 larvae (B-B”,C-C”). For each experiment, the post-bleach frame chosen corresponds to a time point after recovery plateau had been reached. Mean fluorescence intensity measurements were taken within the bleach ROI (white box) and, in L1 experiments, an adjacent ROI (purple box). Scale bars, 5 μm. (D-F) Fluorescence recovery curves plotting mean and SE for n≥5 replicates per stage for ssSfGFP::LET-653 and n = 2 replicates for ssSfGFP::(PAN). t = 0s represents the first post-bleach frame; raw values for each experiment were normalized to the highest measurement taken. (G, H) Comparisons of mobile fractions and recovery half-times. Full-length LET-653 (D,E) exhibited a significantly lower average mobile fraction than LET-653(PAN) (F). However, the half-time of recovery (t_1/2_) was not significantly different between the two fusion proteins. ** p<0.01, * p<0.05, Student’s *t*-test, two-tailed.

**Fig 11:**
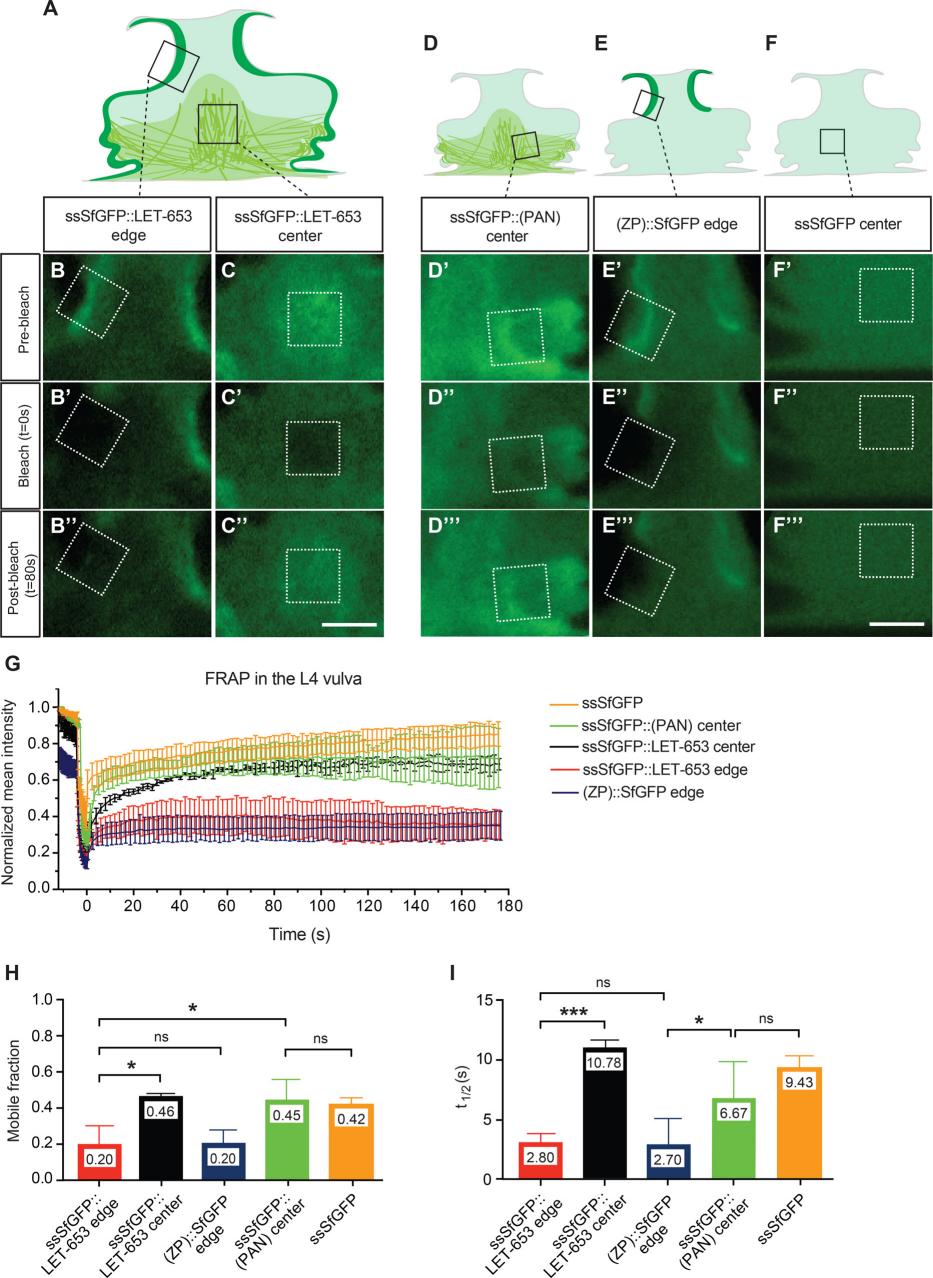
The LET-653 C-terminus remains stably associated with the ZP-containing membrane-proximal matrix compartment. (A-C) FRAP on two spatially distinct pools of full-length LET-653 in the L4 larva vulval lumen. (A) Schematic of ssSfGFP::LET-653 localization in the vulva. Boxes represent bleach ROIs shown as dotted white boxes in (B-B”,C-C”). Pre-bleach, bleach, and post-bleach frames taken from experiments on edge-associated (B) and central (C) compartments. Recovery of the edge region is minimal. (D-F) FRAP on ssSfGFP::LET-653(PAN) (D-D”), LET-653(ZP)::SfGFP (E-E”), or ssSfGFP alone (F-F”). Scale bars, 5 μm. (G) Fluorescence recovery curves plotting mean and SE for n≥2 replicates per genotype and region. (H, I) Comparisons of mobile fractions and recovery half-times. ZP-containing fusions in the edge domain are significantly less mobile than fusions in the central domain, irrespective of genotype or tag placement. Faster recovery in the edge experiments may be attributed to fast-moving protein in the adjacent diffuse pool, some of which was included in the ROIs chosen (B, E’). *** p<0.001, * p<0.05, Student’s *t*-test, two-tailed.

ZP-containing fibrillar matrices typically are quite stable [25]. To test the stability of LET-653-mediated interactions, we assessed the mobility of LET-653 fusions using fluorescence recovery after photobleaching (FRAP) experiments. We performed these experiments in the duct lumen at two different developmental stages (1.5-fold and late L1) (Fig 10, S4 Fig), and also in the L4 vulva, where we could compare apical membrane-localized vs. centrally-located pools of LET-653 (Fig 11). In the duct lumen, mobility of the full-length ssSfGFP::LET-653 fusion decreased between the 1.5-fold and the L1 stages (Fig 10D and 10G), and at L1, the mobility of this fusion was significantly lower than that of ssSfGFP::LET-653(PAN) (Fig 10E–10G). These data indicate that PAN-mediated interactions are relatively dynamic. Furthermore, although the ZP domain is not sufficient to confer duct localization at the L1 stage, it does contribute to more stable interactions. In the vulva, the apical membrane-associated pool of full-length LET-653 was significantly less mobile than the centrally-located pool of either full-length or LET-653(PAN) (Fig 11A–11D and 11G–11I), again suggesting that ZP-mediated interactions are quite stable, while PAN-mediated interactions are more dynamic.

Because cleavage at the CCS and dissociation of the C-terminal region has been proposed to be essential for ZP fibril formation, we compared the above results to that of a C-terminally tagged LET-653(ZP) fusion to ask if C-terminus retention affects LET-653 mobility. Consistent with earlier results demonstrating that N- and C- terminally tagged LET-653 localized similarly (Fig 5), LET-653(ZP)::SfGFP also localized to the vulva apical membrane and behaved identically to the apical membrane pool of full-length ssSfGFP::LET-653 in FRAP experiments (Fig 11E and 11G–11I).

### LET-653 cooperates with the lipocalin LPR-1 to maintain tube integrity

**Fig 12:**
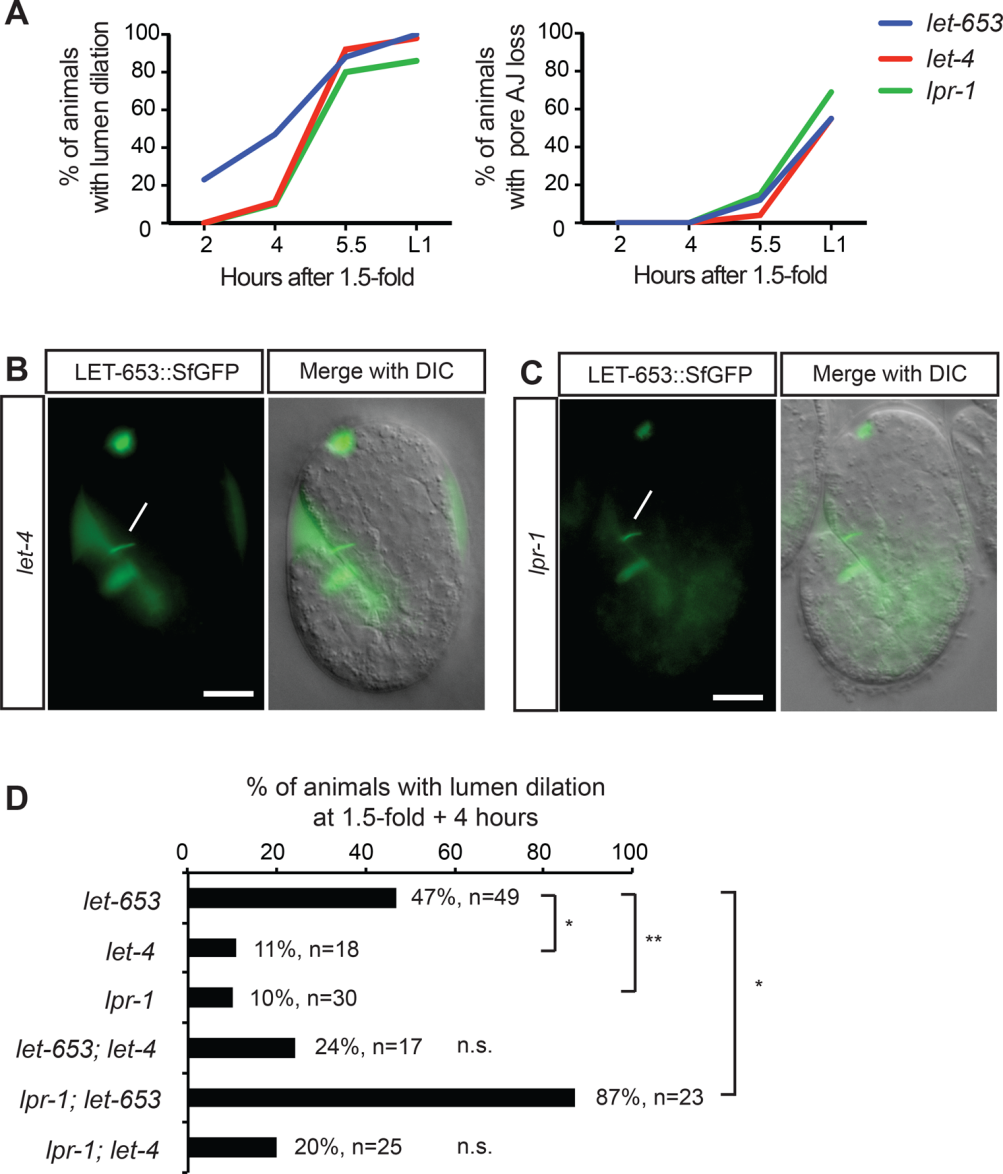
*let-653* functions in parallel to *lpr-1*. (A) Comparative timecourse of lumen dilation and pore AJ loss in *let-4(mn105)*, *lpr-1(cs73)*, and *let-653(cs178)* mutants. *let-653* single mutant data are reproduced from Fig 2A. n at least 17 animals per genotype for each timepoint. (B) *let-4(mn105)* and (C) *lpr-1(cs207)* embryos bearing the LET-653::SfGFP transgene. LET-653::SfGFP is visible in the duct/pore lumen (lines). Scale bars, 10 μm. (D) Double mutant analysis. ** p<0.002, *p<0.01, Fisher’s Exact test, compared to single mutants at same timepoint.

The *let-653* duct lumen fragmentation and G1 pore junction loss phenotypes are very similar to those caused by loss of the apical eLRRon protein LET-4 [38] or the secreted lipocalin LPR-1 [45]. However, lumen abnormalities appeared somewhat later in the other mutants compared to *let-653* (Fig 12A). Loss of *let-4* or *lpr-1* did not perturb LET-653::SfGFP apical secretion or duct luminal retention in embryos (Fig 12B and 12C).

To test the functional relationship between *let-653* and *let-4* or *lpr-1*, we analyzed double mutants (Fig 12D). At an early timepoint where each single mutant had lumen defects at low to moderate penetrance, *let-653; let-4* or *lpr-1; let-4* double mutants were not significantly worse than single mutants. In contrast, *lpr-1; let-653* double mutants displayed a significantly more penetrant lumen phenotype. These data suggest that *let-653* and *lpr-1* act in parallel and cooperate to maintain lumen integrity at early stages, while *let-4* may act at a later step of cuticle matrix assembly.

## Discussion

There is increasing recognition of the importance and disease relevance of the aECM or glycocalyx, which lines the apical surfaces of epithelia and the luminal surfaces of cellular tubes. Nevertheless, the aECM has been much less studied than the basal ECM, in part due to challenges in visualizing the aECM both *in vivo* and *ex vivo*. Relatively little is known about the mechanisms underlying aECM functions, how different components of the aECM interact with each other and with the apical membrane, and how the aECM is assembled and cleared. This work identifies the PAN-Apple, mucin and ZP domain glycoprotein LET-653 as a component of a transient apical glycocalyx that precedes cuticle secretion in *C*. *elegans*. Although LET-653 is broadly expressed and affects apical domain shaping of multiple epithelial tissues, including the epidermis and vulva, it is particularly critical to maintain patency and integrity of the very narrow unicellular excretory duct and pore tubes. Different domains within LET-653 localize the protein to distinct matrix pools, and ZP-dependent interactions near the apical membrane are critical for lumen shaping and integrity during a period of dramatic tube elongation and narrowing.

### LET-653 localizes transiently to two compartments within the pre-cuticular apical glycocalyx

The cuticle or exoskeleton of invertebrates is a particularly prominent example of apical ECM. In *C*. *elegans*, the mature cuticle consists primarily of collagens, but also includes various other insoluble proteins, termed cuticulins, several of which contain a ZP domain [40,50]. The mature cuticle is a multi-layered structure that is built via sequential secretion of different matrix components, and it has been presumed that the earliest secreted layers end up on the most apical surface [69,71]. The earliest observed layer, the embryonic sheath, forms between the bean and 1.5-fold stages [60]. We showed that LET-653 is secreted apically prior to formation of the embryonic sheath, but then disappears prior to the bulk of cuticle secretion. We found no evidence that LET-653 persists as part of the epicuticle. However, LET-653 absence does affect epidermal cuticle structure and alae morphology, consistent with the idea that the early glycocalyx affects deposition or organization of subsequent matrix layers.

**Fig 13:**
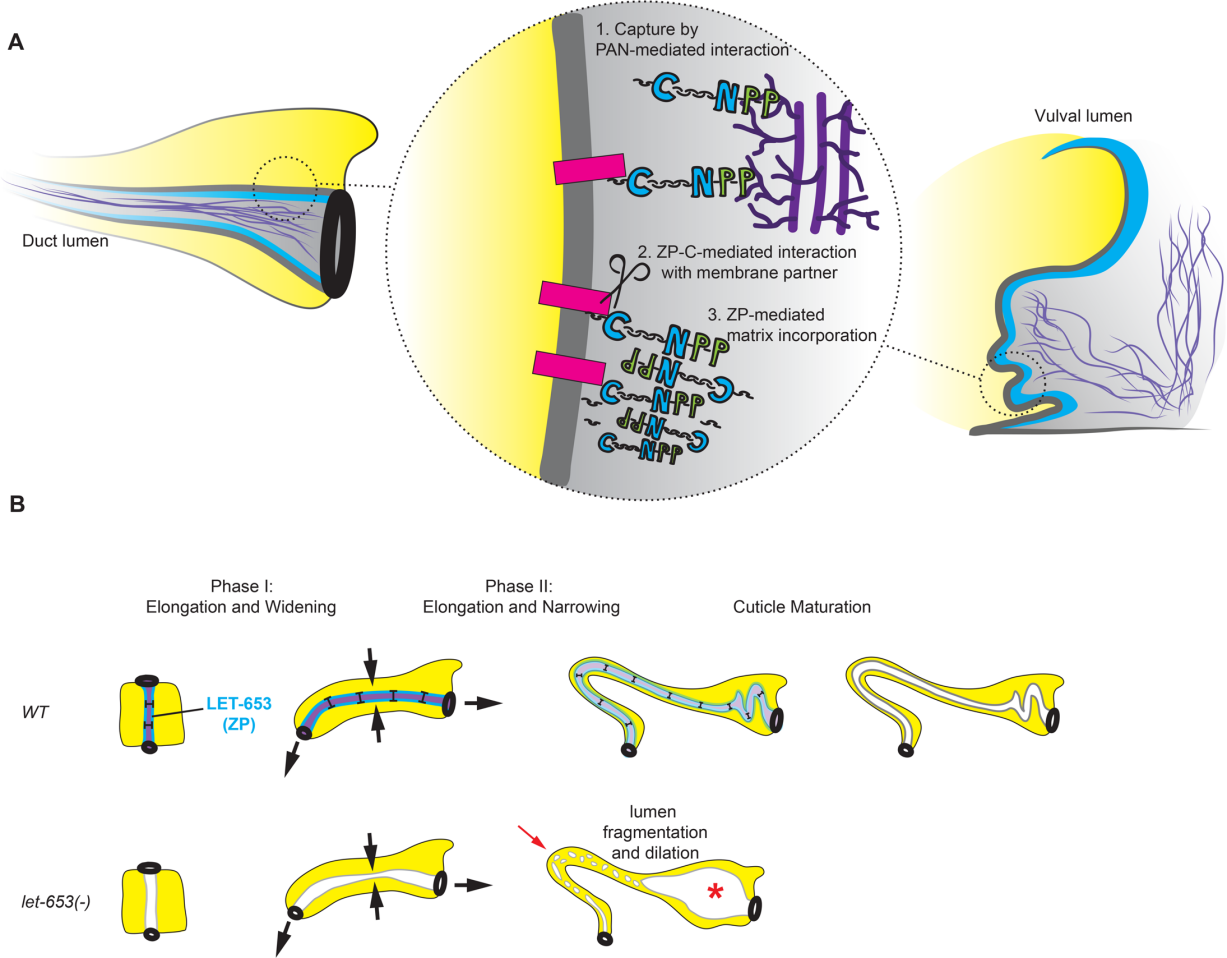
Summary and model for the role of LET-653 in tube morphogenesis. (A) Model for LET-653 aECM assembly. Upon LET-653 secretion, PAN-mediated interactions with the luminal matrix (purple) capture LET-653 and prevent its rapid outflow from the lumen. The ZP-C and/or C-terminal regions then interact with a membrane-associated factor (pink rectangle) that anchors LET-653 more tightly. A membrane-associated protease (scissors) cleaves LET-653 at the CCS, allowing ZP-N dependent polymerization and/or recruitment of additional matrix factors; however, the C-terminal peptide (black squiggle) remains tightly affiliated with the matrix. Arrangement of LET-653 polymers is hypothetical; see text for alternative models. (B) Role of LET-653 in duct tube maintenance. During phase I of duct tube morphogenesis, membrane-associated LET-653(ZP) (blue) resists (T-bars) stretching and/or constrictive forces (arrows) to maintain a uniform lumen diameter and allow fluid passage. During phase II, LET-653 is gradually cleared from the lumen, and a LET-653-dependent cuticle structure begins to form and take over the lumen shaping role. Finally, once tube morphogenesis is complete, the cuticle matures. In *let-653* mutants, the lumen becomes too narrow during elongation and fragments into separate compartments (arrow). Upstream of the fragmentation, the lumen dilates (asterisk) as a secondary consequence of fluid accumulation.

We were able to visualize the early aECM in the vulva by both live confocal imaging and TEM. In this large, multicellular tube, LET-653 localizes to two distinct pre-cuticular aECM compartments (Fig 13A). LET-653 interacts dynamically via its PAN domains with a loose, fibrillar matrix in the center of the lumen, which corresponds roughly to the region where chondroitin proteoglycans (CPGs) are most abundant [72]. LET-653 interacts more stably via its ZP domain with a matrix compartment along the apical membrane, where cuticle assembly will later occur. This compartment may be equivalent to the embryonic sheath in the epidermis, although it is much thicker than the sheath in some regions. At the base of the vulva lumen, the two matrix compartments are connected via LET-653(PAN)-decorated fibrils. The specific partners that LET-653 interacts with in these two compartments remain to be identified, but LET-653 domain-specific reporters will be excellent tools for testing candidate partners.

TEM data [38] and our PAN- and ZP-domain localization data are consistent with a similar two-compartment aECM organization during early excretory duct and pore tube development. The LET-653 PAN domain is both necessary and sufficient to confer a cyclic pattern of localization to the duct/pore lumen, but the ZP domain is not, suggesting that a PAN-mediated interaction with the central luminal matrix captures LET-653, preventing its outflow from the lumen and allowing other ZP-mediated interactions to occur (Fig 13A). The cyclic reappearance and disappearance of LET-653 in the larval duct/pore lumen is apparently not required to maintain tube integrity, but likely reflects other dynamic changes in the matrix environment that occur surrounding each molt. Disappearance of LET-653 from the duct lumen could result from clearance of its relevant binding partners or severing of its connections to those partners.

### Role of the LET-653 ZP domain in lumen shaping

Although LET-653 has often been cited in the literature as an example of mucin-dependent tube shaping (for example, see [5]), we found that the mucin-like region was not sufficient to rescue duct/pore integrity defects in *let-653* mutants, even when tethered in the lumen by the PAN domains. Furthermore, the mucin-like region did not affect LET-653 matrix localization or gut lumen expansion. While these observations do not exclude a relevant role for the mucin-like region, they do show that tube protective activity and lumen expansion require other portions of the LET-653 ZP domain.

The ability of the LET-653 ZP domain to localize to the membrane-proximal matrix compartment correlates absolutely with duct/pore protective activity. Since the duct lumen elongates significantly over the period that LET-653 is present, the LET-653 matrix must be quite flexible or capable of rapid remodeling to accommodate this elongation. Glycosylated mucin domains can form rod-like structures that extend over long distances [73,74], so one possibility is that progressive glycosylation of this domain allows it to stretch. The mechanisms driving duct elongation are not known, but the duct is likely subjected to an external stretching force as it has apical junctions to partners (the canal and pore tubes) that move apart as the embryo elongates, gains motility and begins to flex its body. Such stretching forces and/or additional constrictive forces within the duct cell could contribute to narrowing of the duct lumen. A LET-653 ZP-dependent expansion activity is needed to counteract such narrowing and maintain a patent lumen with a uniform diameter. In *let-653* mutants, the lumen still forms and expands to some degree, but it is irregular in diameter and fragments during the narrowing phase (Fig 13B). Because LET-653 is already disappearing as lumen narrowing occurs (Fig 13B), we propose that the membrane-anchored LET-653 matrix not only directly expands and constrains lumen dimensions during early tube morphogenesis, but also serves as a scaffold for assembly of later cuticle matrix layers that are important for continued lumen integrity.

ZP domains typically confer the ability of proteins to homo- or hetero-polymerize into mesh-like fibrils [49]. The mechanisms that regulate ZP matrix assembly are not well understood, but interactions among the ZP-N, ZP-C and C-terminal subdomains appear to hold the protein in a polymerization incompetent conformation to allow trafficking through the secretory pathway [55–57,68,75]. Once at the plasma membrane, cleavage at a CCS to remove the C-terminus is thought to eliminate those inhibitory interactions to allow polymerization [54,55,57]. Prior to or concomitant with cleavage, interactions between the C-terminus and other factors may also facilitate proper matrix assembly [75].

Our structure/function data indicate that the LET-653 C-terminus is required for proper secretion, C-terminal cleavage does occur, and the region encompassing the ZP-C and C-terminal domains is essential for apical localization and duct protective activity. Therefore, a reasonable model is that the stable pool of LET-653 near the apical membrane consists of ZP-dependent polymers whose localization and anchoring depend on unknown ZP-C- or C-terminal domain interactors (Fig 13A). We note, however, that there is also evidence for non-polymerizing, signaling roles of some luminal ZP-domain proteins, such as mammalian Endoglin and Betaglycan [26,29], as well as for mucins [15–17,76]. Furthermore, LET-653 lacks otherwise conserved aromatic residues in the ZP-N domain that are thought to be important for polymerization (S1 Fig) [77], and the C-terminal domain remains stably associated with the membrane-proximal LET-653 matrix rather than dissociating as expected (Fig 12E). Therefore, other potential models are that LET-653 affects assembly of other matrix proteins or interacts with transmembrane factor(s) to affect signaling and/or cytoskeletal organization.

### Apical ZP-containing matrices and the integrity of narrow tubes

Why is LET-653 essential for duct/pore integrity, whereas it has only modest effects on shaping of larger tubes such as the vulva? The distinction could merely reflect redundancy, if other aECM factors, such as chondroitin proteoglycans, compensate for the absence of LET-653 in some tissues but not others. However, other observations also suggest that narrow tubes may be particularly sensitive to disruptions in ZP-domain components of the aECM. In the Drosophila trachea, loss of the ZP proteins Piopio or Dumpy causes junctions between smaller tubes to break, whereas larger tubes generally remain intact but over-elongate and change shape [24,25,78]. The mammalian microvasculature contains many unicellular capillaries [31], and loss of Endoglin, a luminal ZP protein of endothelial cells, causes capillary ruptures and hemorrhage in both mice and human patients [79,80]. Endoglin phenotypes have been attributed to defects in TGF-beta or BMP signaling, although additional direct effects on physical integrity have not been ruled out [80]. ZP-dependent apical shaping roles could be particularly important in narrow tubes, where apical membranes otherwise could easily come in contact with each other, leading to membrane damage or lumen collapse.

### Relationship among ZP proteins, eLRRon proteins and lipocalins

LET-653 is just one of a set of transmembrane or secreted proteins that have similar requirements in the maintenance of the excretory duct and pore tubes. Others include the apically-localized transmembrane eLRRon proteins LET-4 and EGG-6 [38] and the secreted lipocalin LPR-1 [45]. Our data suggest that LET-653 and LPR-1 act in parallel to promote duct lumen integrity during morphogenesis; cooperative but distinct mechanisms of action are consistent with prior evidence that LPR-1 can protect lumen integrity even when provided from outside of the excretory system [45]. LET-4 appears to affect a later step of aECM organization, with a later onset of expression and broader effects on cuticle organization and barrier function that are not seen in *let-653* mutants [38].

Possible functional relationships among ZP proteins, eLRRon proteins and lipocalins have been found in other systems. In Drosophila, the PAN and ZP domain protein NOMP-A and the eLRRon protein Artichoke are both constituents of the dendritic cap matrix that links ciliated neurons to glial structures within mechanosensory and chemosensory organs [81,82]. Mutations in the ZP proteins Piopio and Dumpy and in the eLRRon proteins Convoluted, Capricious and Tartan all affect tracheal tube shape and/or cell connectivity [24,83,84]. In the mammalian kidney, the ZP domain protein uromodulin (UMOD, also known as Tamm-Horsfall protein) and Lipocalin2 (Lcn2, also known as NGAL or 24p3) are among the most abundant luminal components in nephron tubules, where they appear to play protective functions [27,85]. Mutations in UMOD cause inherited forms of chronic kidney disease (CKD) that may be associated with structural damage to nephron tubules [27,86]. Further studies of ZP, eLRRon and lipocalin proteins in the *C*. *elegans* excretory system should better our understanding of the molecular and cellular roles of these families of apical factors, and how disruptions of aECM organization can contribute to disease.

## Materials and Methods

### Worm strains, alleles and transgenes

All strains were grown at 20˚C under standard conditions [87] unless otherwise noted. Allele *let-653(s1733)* [42] is linked to *unc-22(s7)* and *unc-31(e169)*. Alleles *let-653(cs178)* and *let-653(cs204)* are linked to *jcIs1*. Mutants were obtained from mothers heterozygous for the balancer *nT1 [qIs51]* [88] or rescued with a *let-653(+)* transgene. Transgenes used included: *jcIs1* (AJM-1::GFP, *rol-6d*) IV [89], *qnEx59 (dct-5pro*::mCherry, *unc-119+*) [46], *sEx10642* (*let-653pro*::GFP, *dpy-5+*) [90]. See S1 and S2 Tables for a complete list of strains and newly generated transgenes used in this study.

### *let-653* mutant isolation and allele identification

Strain UP2214 [*unc-119; jcIs1*; *qnEx59]* was mutagenized with ethylmethanesulfonate (EMS) as described [87], 2239 F1 animals were picked to individual plates and F2 progeny were screened for rod-like lethality. A total of 85 recessive rod-like lethal mutations were identified, of which 61 had obvious duct or pore junction or lumen abnormalities. Of 24 mutants with initially WT junction patterns as embryos, two mapped to chromosome IV and failed to complement *let-653(s1733)* and each other. Lesions were identified by whole genome sequencing followed by bioinformatics analysis with Cloudmap [91] and/or by Sanger sequencing of PCR-amplified genomic fragments. *cs178* changes codon 54 from CAA to TAA. *cs204* changes the intron 4 splice acceptor from CAG to CAA. *s1733* changes codon 250 from TGT to TGA. All three *let-653* alleles also contain a common polymorphism (a CCG to CTG change at codon 189, leading to a P189L substitution) that differs from the reference N2 and cDNA sequence.

### Plasmids

For tissue-specific rescue experiments, *let-653b* cDNA was PCR-amplified from yk1667d04 with primers oCP10 (GGGGCTAGCAAAATGCGACATCCACTAATTTCTCTAC) and oCP2 (GGGGGTACCTCAGATGTTTCCAGTTCGAAC) and cloned into derivatives of vector pPD49.26 (Addgene) containing different tissue-specific promoters. *lpr-1pro* drives expression in the duct, pore and hypodermis beginning at the 1.5-fold stage [59]. *lin-48pro* drives expression in the duct cell beginning at the 2-fold stage [92]. *dpy-7pro* drives expression in the pore and hypodermis beginning at the 1.5-fold stage [93]. *glt-3pro* drives expression in the canal cell beginning at the early 3-fold stage [94]. *unc-54pro* drives expression in the body muscle beginning at the 1.5-fold stage [95]. Plasmids were co-injected with marker pIM175 *unc-119pro*::*GFP* (see S1 Table).

For localization and structure/function experiments, a 2.2 kb fragment containing the *let-653* promoter and upstream regulatory region was PCR-amplified from the *let-653* TransgeneOme clone [96] with primers oJAF4 (GGGAAGCTTGTCTATGTGAACCAGTCAATG) and oJAF5 (GGGGGATCCGTGGATGTCGGATTTACTGAAGAGAGCAG) and cloned into pPD49.26, upstream of tagged *let-653b* cDNAs. See S2 Table for a complete list of LET-653 full-length fusions and structure/function constructs. SfGFP was obtained from pCW11 (a kind gift from Max Heiman, Harvard U.). Plasmids were co-injected with marker pHS4 (*lin-48pro*::mRFP) (see S1 Table). Animals transgenic for the TransgeneOme LET-653::GFP reporter [96] showed a similar pattern of GFP expression and localization to that reported in Fig 5, but expression appeared much fainter.

### Staging and microscopy

Embryos were selected at the 1.5-fold stage and then incubated for the indicated number of hours before mounting for imaging. Larvae were staged by hours after egg-lay, with five hours corresponding to the 1.5-fold stage and thirteen hours corresponding to hatch.

Fluorescent and DIC images were captured on a compound Zeiss Axioskop fitted with a Leica DFC360 FX camera or with a Leica TCS SP8 confocal microscope. Images were processed and merged using ImageJ and Adobe Photoshop. Note that there is bleed-through of the red channel into the green channel in some Axioskop images, so many images are displayed as inverted grayscale with both channels visible. Duct lumen dimensions were measured using the line tool in ImageJ. For each specimen, measurements were made in triplicate and then averaged.

For TEM, the *let-653(s1733)* L1 specimens were cut open with a razor blade before fixation in buffered glutaraldehyde, rinsed and fixed again in buffered osmium tetroxide, then washed, en bloc stained with uranyl acetate, dehydrated and embedded into plastic resin [37,97]. The wild-type L1 specimen (called “L1C”) shown in Figs 3B, 3C, and 4C and the wild-type L4 specimen (“N2_L4_vulva”) shown in Fig 7A did not have a primary fix in glutaraldehyde, but were just fixed in buffered osmium tetroxide before dehydration and embedment. The normal (*lin-17*) and *let-653(cs178)* embryo specimens in Fig 3A and 3D were prepared by high pressure freezing and freeze substitution into osmium tetroxide in acetone, then rinsed and embedded into LX112 resin [98]. Images were collected on a Jeol-1010 transmission electron microscope, processed in ImageJ and pseudocolored in Adobe Illustrator.

### Western blotting

Worms were grown to near-confluence on 60mm plates. Two 60mm plates were bleached and embryos were allowed to develop in M9 for 4–5 hours until they reached 2-fold stage. Worms were pelleted and transferred to Laemmli buffer (BioRad, 161–0737) with 1x Protease Inhibitor Cocktail (Sigma, 2714) and 1:20 B-mercaptoethanol. Samples were boiled for 5 minutes and then transferred to -80C until ready for use. Samples were boiled for 10 minutes before loading into Mini-Protean TGX gradient gels (BioRad, 456–1084). Electrophoresis was performed at 0.03A-0.04A under 1x electrophoresis buffer (BioRad, 161–0732). Protein was transferred onto 0.2um nitrocellulose membrane (BioRad, 162–0147) overnight in 1x transfer buffer (20% ethanol, 0.58% Tris Base, 2.9% Glycine, 0.01% SDS). Membranes were washed in PBS + 0.02% Triton (PBST) and blocked for 1 hour at room temperature in PBST + 1% dry, nonfat milk. Membranes were probed for GFP for 1 hour at room temperature with PBST + 1% milk + 1:500 anti-GFP antibody (Rockland Immunochemicals, 600-101-215). Membranes were washed in PBST and then probed with PBST + 1% milk + 1:2000 anti-Goat-HRP antibody (Rockland Immunochemicals, 605–4302) for 1 hour at room temperature. Membranes were washed with PBST before detection using SuperSignal West Femto Maximum Sensitivity Substrate (Pierce 34095) under a CCD camera (AV Imaging Systems). For the loading control, membranes were stripped for 15 minutes in Restore Western Blot Stripping Buffer (Thermo Scientific, 21059) and then washed in PBST. The same protocol was used for the loading control as for the primary blot. Anti-UNC-15/paramyosin (R224, kindly provided by Dr. Jeff Hardin, Univ. of Wisconsin) diluted 1:2000 was used as the primary antibody and anti-rabbit IgG (GE Healthcare, NA934V) diluted 1:10,000 was used as the secondary antibody.

### Fluorescence recovery after photobleaching (FRAP)

Specimens were mounted on 10% agarose pads containing 20mM sodium azide and 10mM levamisole in M9. FRAP was performed using Leica Application Suite X software FRAP module on a Leica TCS SP8 MP confocal microscope. For 1.5-fold embryos and L1 larvae, a 1 μm X 1 μm bleach ROI was defined within the wizard, and mean fluorescence intensity measurements within the ROI were taken at specified intervals. The following experimental time-course was used: 20 pre-bleach frames every 0.4 seconds, 5 bleach frames every 0.4 seconds, and 90 post-bleach frames every 2.0 seconds. Laser intensity during bleach was set to 70.0%, while pre- and post-bleach laser intensity varied from 0.25% to 1.50% on a specimen-by-specimen basis. A pinhole size of 3.0 (units) was used for all FRAP experiments. For L4 larvae, bleach laser intensity was increased to 100%, the number of bleach frames was increased to 10, and the ROI size was increased to 3 μm X 3 μm.

FRAP plots were created and analyzed in Prism, where one-phase association curves derived from the model Y = Y0 + (Plateau—Y0)*(1 –e^(-Kx)) were fitted to the data. For statistical tests, mobile fractions and recovery half-times were derived from one-phase association curves fitted to individual experiments. Mobile fraction = Plateau-Y0; t_1/2_ = ln(2)/K, where K is the recovery rate constant.

### Heat shock experiments

Full-length or partial *let-653b* cDNAs, tagged with SfGFP or ssSfGFP were cloned into the *hsp16*.*41* promoter-containing vector pPD49.78 (Addgene) (S2 Table). Transgenic embryos were heat shocked at 34°C for 30 minutes 3 hours after egg lay and imaged 2.5 hours later. Gut lumen width measurements were taken at the widest point using ImageJ freehand line tool and Plot Profile function.

## Supporting Information

S1 Fig Complete LET-653b protein sequence. Additional data related to Fig 1. (A) Annotated protein sequence showing domains and sequence features of interest. Known N-glycosylation sites are from [51]. (B) LET-653 lacks conserved aromatic residues involved in ZP polymerization. Alignment of a portion of the ZP-N domain from LET-653 with the corresponding regions of *C*. *elegans* DYF-7 (NP_509630.1), Drosophila Dumpy (AGB92578.1), and human ZP3 (NP_001103824.1), uromodulin (NP_003352.2), tectorin alpha (NP_005413.2) and endoglin (NP_001108225.1). Grey highlights indicate beta strands, as defined by [56] or predicted by Predictprotein [102]. Blue highlights conserved cysteines. Red highlights other conserved residues present in polymerizing proteins but missing in LET-653 and endoglin, a known non-polymerizing ZP protein.
(TIF)

S2 Fig *let-653* is expressed in external epithelia. Additional data related to Fig 5. (A, B) *let-653pro*::GFP reporter *sEx10642* expression in the major hypodermis (hyp) at ventral enclosure (A) and in the excretory duct and pore at the 3-fold stage (B). Expression is only occasionally and transiently observed in the canal cell. A’ and B’ show DIC images for comparison. (E) LET-653b::SfGFP driven by the canal-specific *glt-3* promoter occasionally accumulates in the duct lumen of embryos. (F) LET-653b::SfGFP driven by the non-cycling *lin-48* promoter also shows an oscillatory pattern of accumulation in the duct lumen. n>15 animals for each timepoint. (G-L) Both *let-653pro*::LET-653::SfGFP and *let-653pro*::ssSfGFP::LET-653 reveal apical secretion and accumulation in extracellular and luminal regions. (G, J) ventral enclosure embryos. (H, K) 3-fold embryos. E’,G’,H’,I’,J’ show merged DIC images for comparison. (I, L) Both LET-653::SfGFP and ssSfGFP::LET-653 are absent from the early L1 duct cell lumen, after mature cuticle secretion and hatch. K’ and L’ show absent SfGFP signal. ssSfGFP::LET-653 appears within the space between the old and new cuticles during the L1/L2 molt (M,N). M’ and N’ show DIC images for comparison. Scale bars, 10 μm.
(TIF)

S3 Fig The LET-653 ZP-C domain is required for duct protective activity and apical membrane localization. Additional data related to Fig 8. (A-B) LET-653 fusions truncated before (A) or after (B) the mucin-like domain do not rescue *let-653* mutant lethality. (C,D) Both fusions localized normally to the duct in 1.5 fold embryos. (E,F) Both fusions accumulated in the late L1 larval duct lumen. Confocal slices. (E’, F’) *lin-48pro*::mRFP marks the duct cell. (G,H) Both fusions associated with fibrous material in the center of the vulva lumen, but did not localize to the apical membrane. C’, D’, G’, H’ show DIC images for comparison. Scale bars, 5 μm.
(TIF)

S4 Fig FRAP comparison of N- and C-terminally tagged LET-653 fusions. Additional data related to Fig 10. (A-A”,B-B”,C-C”,D-D”) Example images from FRAP experiments in the excretory duct at 1.5-fold stage (A-A”,B-B”) and L1 (C-C”,D-D”). Bleach ROI shown as dotted white box. Scale bars, 5 μm. (E,F) Fluorescence recovery curve showing mean and SE, n≥5. (G,H) Comparisons of mobile fractions and recovery half-times. (G) At both stages, the mobile fraction was significantly greater for LET-653::SfGFP than for ssSfGFP::LET-653, as expected if some C-terminal tag was cleaved off and released (*, p<0.05, **, p<0.01 Student’s *t*-test, two-tailed). (H) Recovery half-times did not differ between the fusions.
(TIF)

S1 Table Strains, transgenes and alleles. (TIF)

S2 Table Plasmids. (TIF)
