# Supplementary figures and images for: Integrity of Narrow Epithelial Tubes in the C. elegans Excretory System Requires a Transient Luminal Matrix
Source: PLoS Genet. 2016 Aug 2;12(8):e1006205. doi: 10.1371/journal.pgen.1006205 (PMC4970718; doi:10.1371/journal.pgen.1006205)

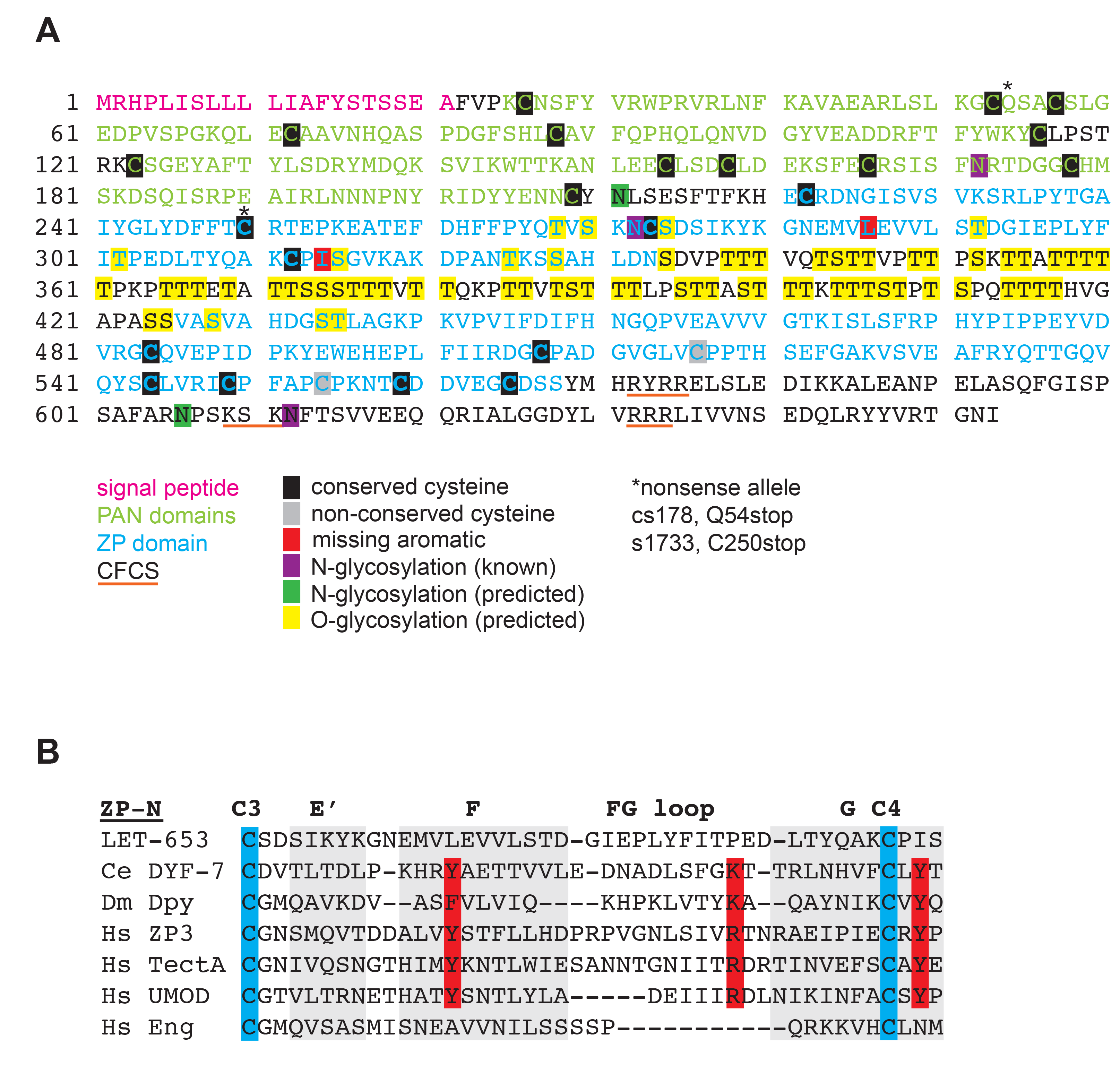

Supplement: S1 Fig — Additional data related to Fig 1. (A) Annotated protein sequence showing domains and sequence features of interest. Known N-glycosylation sites are from [51]. (B) LET-653 lacks conserved aromatic residues involved in ZP polymerization. Alignment of a portion of the ZP-N domain from LET-653 with the corresponding regions of C. elegans DYF-7 (NP_509630.1), Drosophila Dumpy (AGB92578.1), and human ZP3 (NP_001103824.1), uromodulin (NP_003352.2), tectorin alpha (NP_005413.2) and endoglin (NP_001108225.1). Grey highlights indicate beta strands, as defined by [56] or predicted by Predictprotein [102]. Blue highlights conserved cysteines. Red highlights other conserved residues present in polymerizing proteins but missing in LET-653 and endoglin, a known non-polymerizing ZP protein. (TIF) [file pgen.1006205.s001.tif]

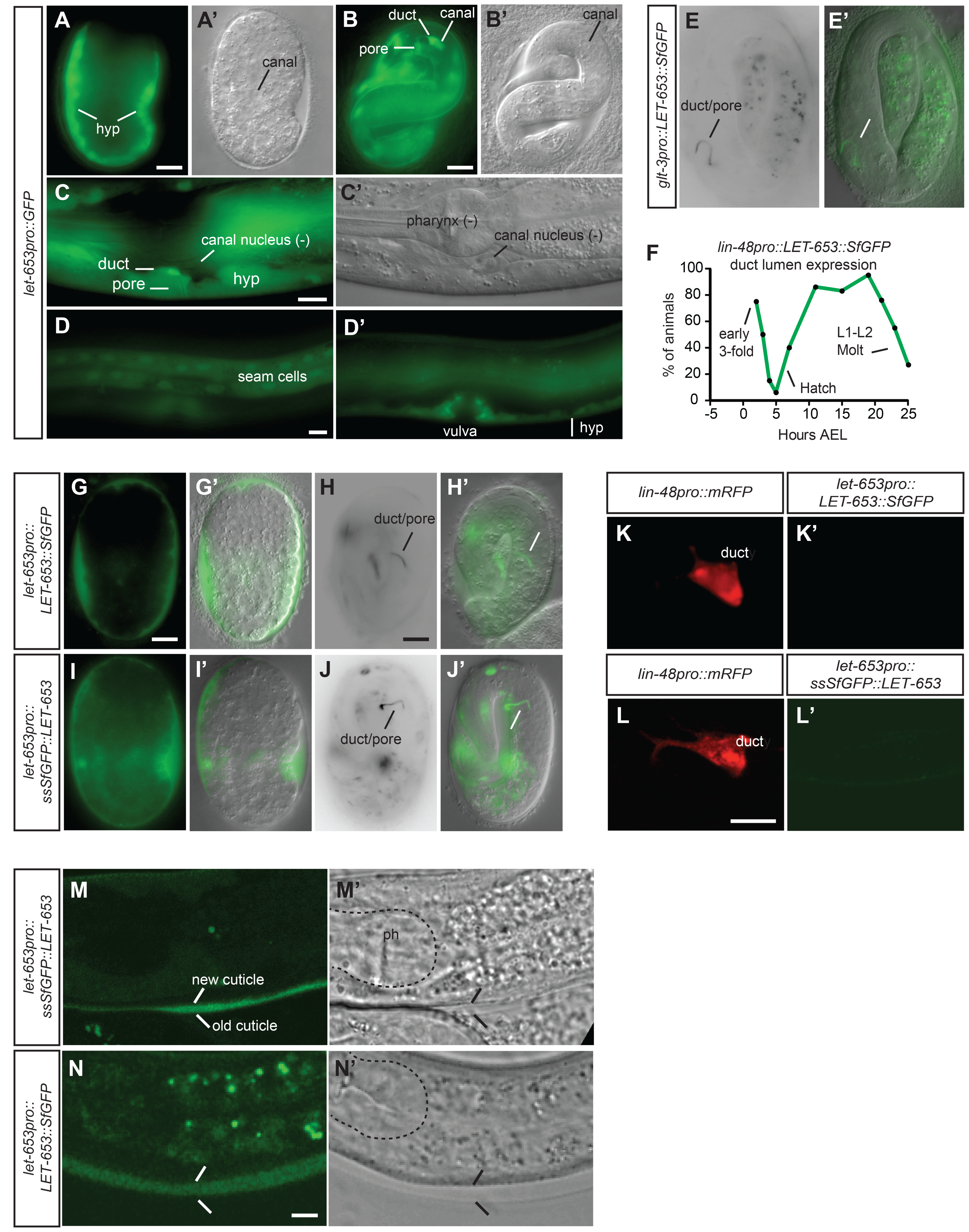

Supplement: S2 Fig — Additional data related to Fig 5. (A, B) let-653pro::GFP reporter sEx10642 expression in the major hypodermis (hyp) at ventral enclosure (A) and in the excretory duct and pore at the 3-fold stage (B). Expression is only occasionally and transiently observed in the canal cell. A’ and B’ show DIC images for comparison. (E) LET-653b::SfGFP driven by the canal-specific glt-3 promoter occasionally accumulates in the duct lumen of embryos. (F) LET-653b::SfGFP driven by the non-cycling lin-48 promoter also shows an oscillatory pattern of accumulation in the duct lumen. n>15 animals for each timepoint. (G-L) Both let-653pro::LET-653::SfGFP and let-653pro::ssSfGFP::LET-653 reveal apical secretion and accumulation in extracellular and luminal regions. (G, J) ventral enclosure embryos. (H, K) 3-fold embryos. E’,G’,H’,I’,J’ show merged DIC images for comparison. (I, L) Both LET-653::SfGFP and ssSfGFP::LET-653 are absent from the early L1 duct cell lumen, after mature cuticle secretion and hatch. K’ and L’ show absent SfGFP signal. ssSfGFP::LET-653 appears within the space between the old and new cuticles during the L1/L2 molt (M,N). M’ and N’ show DIC images for comparison. Scale bars, 10 μm. (TIF) [file pgen.1006205.s002.tif]

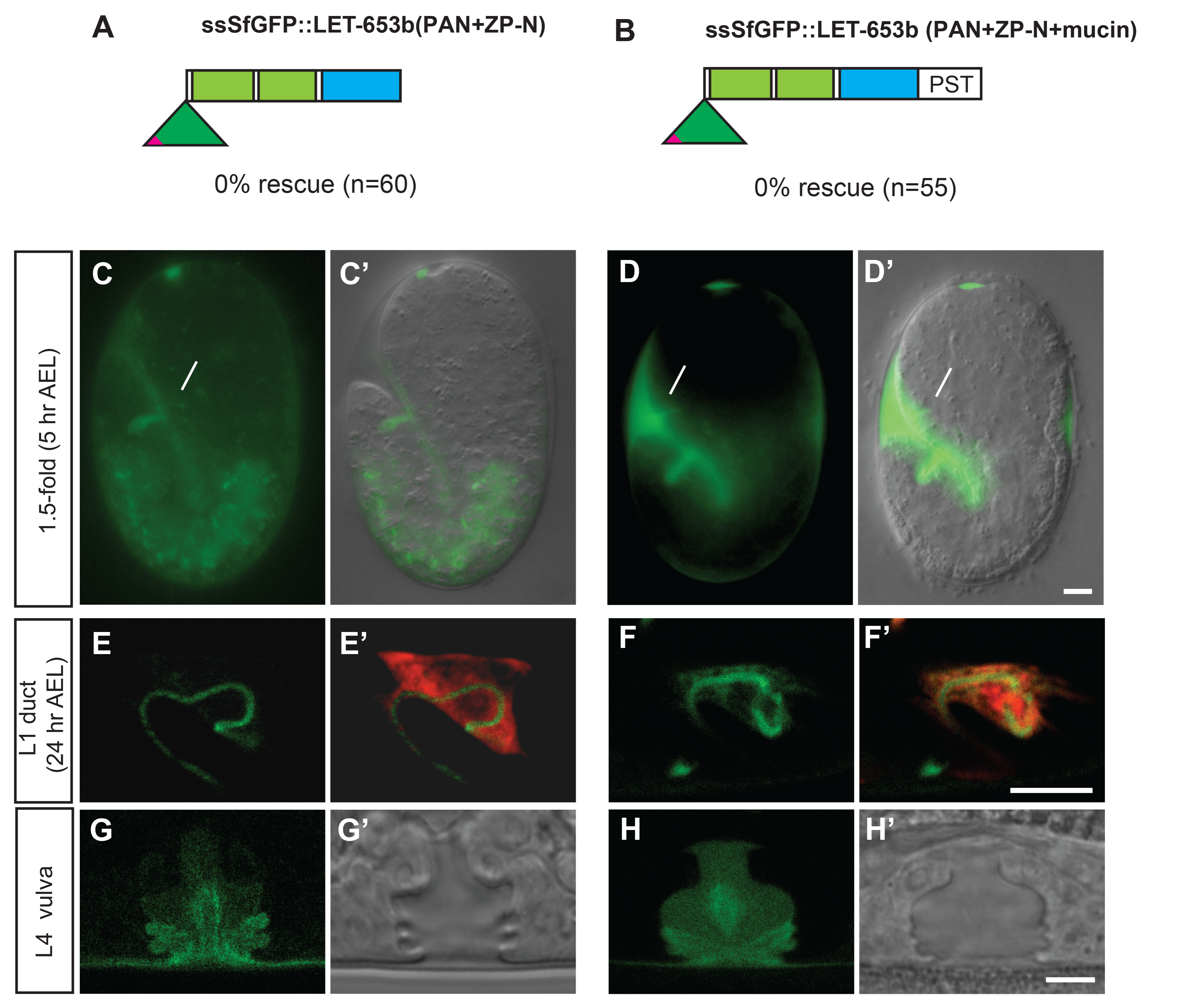

Supplement: S3 Fig — Additional data related to Fig 8. (A-B) LET-653 fusions truncated before (A) or after (B) the mucin-like domain do not rescue let-653 mutant lethality. (C,D) Both fusions localized normally to the duct in 1.5 fold embryos. (E,F) Both fusions accumulated in the late L1 larval duct lumen. Confocal slices. (E’, F’) lin-48pro::mRFP marks the duct cell. (G,H) Both fusions associated with fibrous material in the center of the vulva lumen, but did not localize to the apical membrane. C’, D’, G’, H’ show DIC images for comparison. Scale bars, 5 μm. (TIF) [file pgen.1006205.s003.tif]

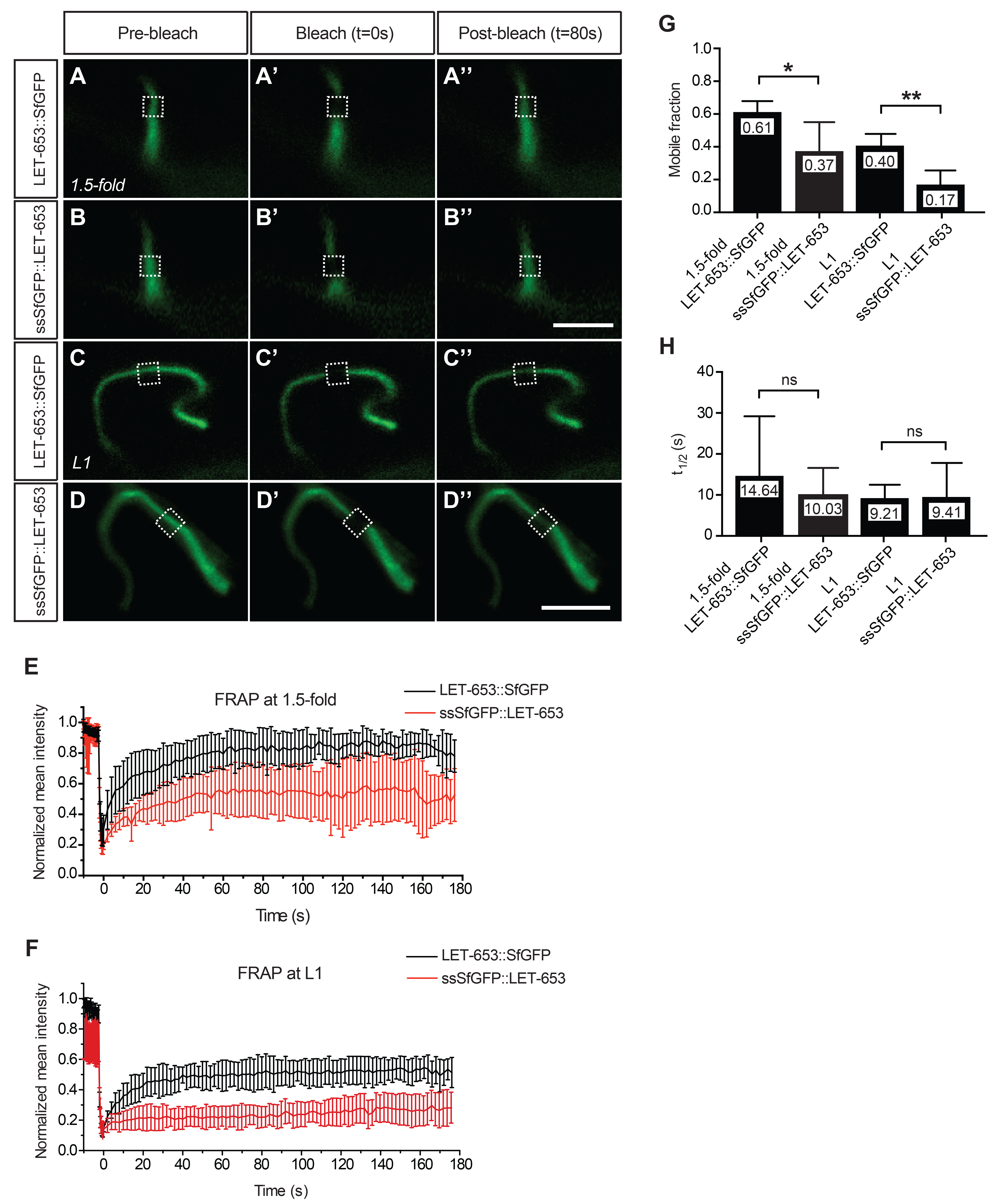

Supplement: S4 Fig — Additional data related to Fig 10. (A-A”,B-B”,C-C”,D-D”) Example images from FRAP experiments in the excretory duct at 1.5-fold stage (A-A”,B-B”) and L1 (C-C”,D-D”). Bleach ROI shown as dotted white box. Scale bars, 5 μm. (E,F) Fluorescence recovery curve showing mean and SE, n≥5. (G,H) Comparisons of mobile fractions and recovery half-times. (G) At both stages, the mobile fraction was significantly greater for LET-653::SfGFP than for ssSfGFP::LET-653, as expected if some C-terminal tag was cleaved off and released (*, p<0.05, **, p<0.01 Student’s t-test, two-tailed). (H) Recovery half-times did not differ between the fusions. (TIF) [file pgen.1006205.s004.tif]

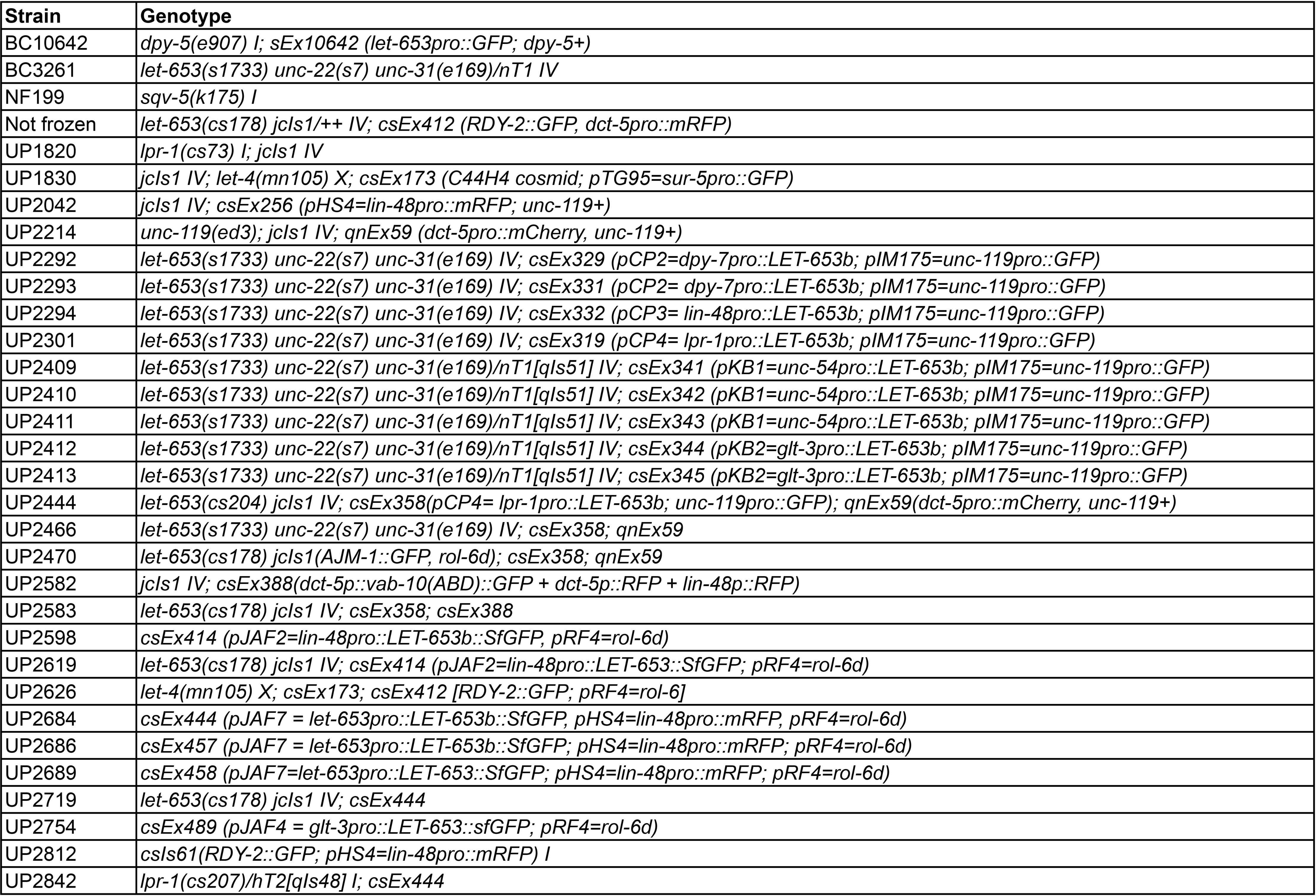

Supplement: S1 Table — (TIF) [file pgen.1006205.s005.tif]

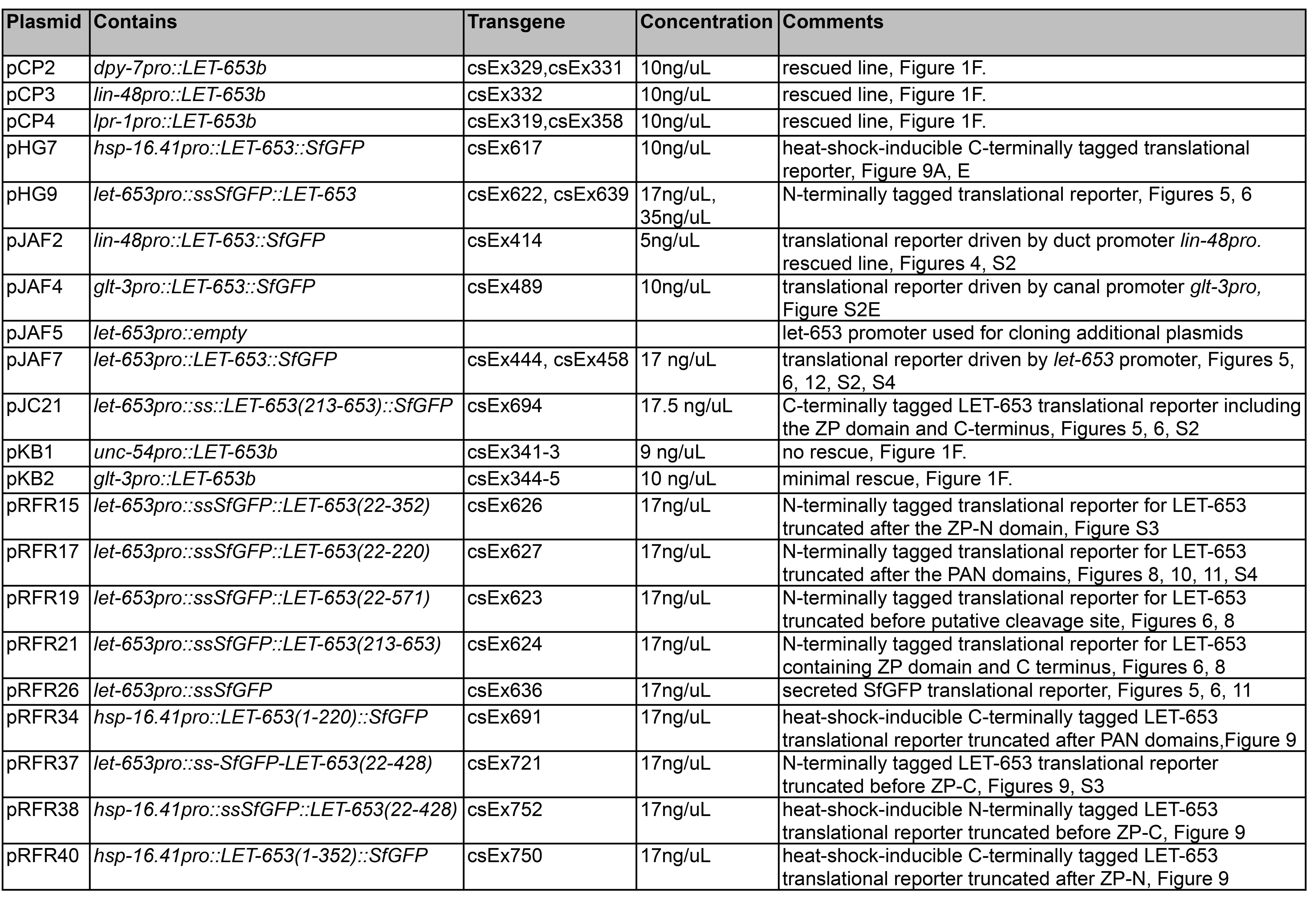

Supplement: S2 Table — (TIF) [file pgen.1006205.s006.tif]
